# Effectiveness of Dry Needling for Myofascial Trigger Points Associated with Neck Pain Symptoms: An Updated Systematic Review and Meta-Analysis

**DOI:** 10.3390/jcm9103300

**Published:** 2020-10-14

**Authors:** Marcos J. Navarro-Santana, Jorge Sanchez-Infante, César Fernández-de-las-Peñas, Joshua A. Cleland, Patricia Martín-Casas, Gustavo Plaza-Manzano

**Affiliations:** 1 Department of Radiology, Rehabilitation and Physiotherapy, Universidad Complutense de Madrid, 28040 Madrid, Spain; marcosjose.navarrosantana@gmail.com (M.J.N.-S.); pmcasas@enf.ucm.es (P.M.-C.); gusplaza@ucm.es (G.P.-M.); 2 Department of Physical Therapy, Rehabilitación San Fernando, 28807 Madrid, Spain; 3 Performance and Sport Rehabilitation Laboratory, Faculty of Sport Sciences, University of Castilla-La Mancha, 45003 Toledo, Spain; jorge.fisio.uclm@gmail.com; 4 Department of Physical Therapy, Occupational Therapy, Physical Medicine and Rehabilitation, Universidad Rey Juan Carlos (URJC), Alcorcón, 28922 Madrid, Spain; 5 Cátedra Institucional en Docencia, Clínica e Investigación en Fisioterapia: Terapia Manual, Punción Seca y Ejercicio Terapéutico, Universidad Rey Juan Carlos, Alcorcón, 28922 Madrid, Spain; 6 Doctor of Physical Therapy Program, Department of Public Health and Community Medicine, Tufts University School of Medicine, Boston, MA 02111, USA; joshcleland@comcast.net; 7 Instituto de Investigación Sanitaria del Hospital Clínico San Carlos, 28040 Madrid, Spain

**Keywords:** dry needling, neck pain, cervical spine, systematic review, meta-analysis

## Abstract

Our aim was to evaluate the effect of dry needling alone as compared to sham needling, no intervention, or other physical interventions applied over trigger points (TrPs) related with neck pain symptoms. Randomized controlled trials including one group receiving dry needling for TrPs associated with neck pain were identified in electronic databases. Outcomes included pain intensity, pain-related disability, pressure pain thresholds, and cervical range of motion. The Cochrane risk of bias tool and the Physiotherapy Evidence Database (PEDro) score were used to assessed risk of bias (RoB) and methodological quality of the trials. The quality of evidence was assessed by using the Grading of Recommendations Assessment, Development, and Evaluation (GRADE) approach. Between-groups mean differences (MD) and standardized mean differences (SMD) were calculated (3) Twenty-eight trials were finally included. Dry needling reduced pain immediately after (MD −1.53, 95% CI −2.29 to −0.76) and at short-term (MD −2.31, 95% CI −3.64 to −0.99) when compared with sham/placebo/waiting list/other form of dry needling and, also, at short-term (MD −0.51, 95% CI −0.95 to −0.06) compared with manual therapy. No differences in comparison with other physical therapy interventions were observed. An effect on pain-related disability at the short-term was found when comparing dry needing with sham/placebo/waiting list/other form of dry needling (SMD −0.87, 95% CI −1.60 to −0.14) but not with manual therapy or other interventions. Dry needling was effective for improving pressure pain thresholds immediately after the intervention (MD 55.48 kPa, 95% CI 27.03 to 83.93). No effect on cervical range of motion of dry needling against either comparative group was found. No between-treatment effect was observed in any outcome at mid-term. Low to moderate evidence suggests that dry needling can be effective for improving pain intensity and pain-related disability in individuals with neck pain symptoms associated with TrPs at the short-term. No significant effects on pressure pain sensitivity or cervical range of motion were observed.

## 1. Introduction

Neck pain is a musculoskeletal condition that often becomes chronic and can result in high levels of disability. The point prevalence is estimated to be 20%, whereas the lifetime prevalence can reach up to 70% in the general population [1]. The Global Burden of Disease Study identified neck pain as the fourth highest condition on number of years lived with disability [2]. Physical therapy is usually the first therapeutic option requested by individuals with neck pain. Several interventions, including cervical manual therapy [3], exercises [4], and education [5], have shown to be effective for the management of neck pain. Clinical practice guidelines for physical therapy management of neck pain recommend manual therapies combined with exercises as the therapeutic strategy for the proper management of these patients [6,7]. Further, clinical practice guidelines do not recommend other treatments, such as dry needling, not because there is evidence against the particular intervention but, rather, there is a lack of studies examining its use.

The etiology of mechanical neck pain is under debate, and it seems to be multifactorial. Some authors proposed that myofascial trigger points (TrPs) can play a role in neck pain development [8]. Simons et al. [8] defined a TrP as “a hypersensitive spot located in a taut band of skeletal muscle which stimulation induces referred pain symptoms and motor phenomena”. There is evidence showing that the referred pain elicited by active TrPs from neck musculature reproduces neck pain symptoms of insidious or traumatic origin [8]. Chiarotto et al. [9] found that TrPs in the upper trapezius is the most common finding in individuals suffering from neck pain. 

Among the several approaches proposed for the treatment of TrPs, dry needling has received particular attention in the last decades [8,10]. Dry needling is defined as a “skilled intervention using a thin filiform needle to penetrate the skin that stimulates myofascial TrPs, muscles, and connective tissue for the treatment of musculoskeletal pain disorders” [11].

A few previous reviews have investigated the effectiveness of dry needling for inactivating TrPs associated with neck pain. Cagnie et al. concluded that dry needling can be recommended for upper trapezius muscle TrPs treatment; however, no quantitative analysis was conducted [12]. Liu et al. concluded that TrP dry needling could be recommended for the management of neck/shoulder pain of myofascial origin at short and mid-term follow-ups [13]. This meta-analysis only included pain intensity as the outcome and considered one month as a mid-term follow-up [13]. In addition, a greater number of randomized clinical trials investigating the effectiveness of dry needling in patients with TrPs associated to neck pain symptoms have been published after the Liu et al. meta-analysis [13]. Therefore, an updated quantitative analysis of the available literature comparing the effects of dry needling vs. sham, control, or other interventions could help to further elucidate its effectiveness. The current updated meta-analysis compares the effects of dry needling against sham, control, no intervention, or other physical therapy interventions applied over muscle TrPs associated with neck pain symptoms on pain intensity, pain-related disability, pressure pain sensitivity, and cervical range of motion. 

## 2. Experimental Section

This systematic review and metanalysis was conducted following the Preferred Reporting Items for Systematic Reviews and Meta-Analyses (PRISMA) statement [14]. The international Open Science Framework Registry link is https://doi.org/10.17605/OSF.IO/P2UWD.

### 2.1. Systematic Literature Search 

An electronic literature search on MEDLINE, CINAHL, PubMed, PEDro, Cochrane Library, SCOPUS, and Web of Science databases was conducted from their inception to 15 July 2020. Searches were restricted to randomized clinical trials, if permitted. The reference lists of the identified papers in database searches were also searched. All search strategy was conducted with the assistance of an experienced librarian.

Population: Adults (older than 18 years) with TrPs in the cervical musculature associated with neck pain symptoms of musculoskeletal origin. 

Intervention: Dry needling of muscle or tendon. Acupuncture was excluded. 

Comparator: Acceptable comparators were any sham or placebo dry needling, any control group without intervention, or any other type of physiotherapy intervention. Interventions should be applied in isolation (self-stretching was permitted).

Outcomes: Pain intensity OR pain-related disability were considered as the primary outcomes. Secondary outcomes included pressure pain thresholds OR cervical range of motion.

The search strategy for each database is available in Table 1.

### 2.2. Selection Criteria

Randomized clinical trials including at least one group receiving any form of dry needling alone in people with myofascial TrPs associated with neck pain were included in the meta-analysis. Since there is no consensus in the terminology, the diagnoses usually associated with TrPs were considered: mechanical/idiopathic neck pain, myofascial neck pain, myofascial pain syndrome, or whiplash-associated disorders.

The following inclusion criteria were considered: (1) adults older than 18 years old with at least at one active TrP in the cervical muscles associated with neck pain symptoms; (2) one group receiving muscle/tendon dry needling; (3) one comparative group including sham or placebo, a control group without intervention, or other physiotherapy intervention; and (4) neck pain intensity or pain-related disability as one of the primary outcomes of the study. Secondary outcomes included sensitivity to pressure pain (e.g., pressure pain thresholds) or cervical range of motion (e.g., as assessed with a goniometer). Exclusion criteria were: (1) trials including participants with neurological-related pain (e.g., post-stroke pain); (2) postoperative cervical pain; (3) trials not published as a full-text journal article; (4) retrospective designs or pilot studies; or (5) the use of needling interventions different than dry needling, e.g., acupuncture or wet needling (e.g., lidocaine injection).

### 2.3. Screening, Selection Process, and Data Extraction

Two authors reviewed the articles identified on each database for their inclusion. After removing duplicates, titles and abstracts of the remaining were screened. Finally, a full-text read of the eligible studies was conducted to determine the inclusion of the trial. The inclusion of a trial was done by consensus between both authors. If discrepancy existed, a third author participated in the process to get a consensus. 

Data including study design, number of subjects, population, interventions, outcome measures, and follow-ups were extracted independently by 2 authors using a specific extraction form. Data extraction was also conducted by consensus. If disagreement occurred, a third author participated.

### 2.4. Assessment of Methodological Quality and Risk of Bias 

The Cochrane Risk of Bias (RoB) assessment tool [15] and the Physiotherapy Evidence Database (PEDro) scale [16] were used to assess the risk of bias and methodological quality of the trials included in the meta-analysis. Methodological quality and RoB were independently assessed by two authors. 

The RoB evaluated the selection bias, performance bias, detection bias, attrition bias, reporting bias, and other bias [15]. Each item was classified as low-risk, high-risk, or unclear according to the Cochrane Collaboration’s tool [15]. The PEDro score evaluated the methodological quality of a trial by assessing the random/concealed allocation, between-groups similarity at baseline, participant/ therapist/assessor blinding, dropouts, intention-to-treat analysis, between-groups comparison, point measures, and variability data [16]. A trial was considered of high quality when the PEDro score was ≥6 out of 10 points.

### 2.5. Level of Evidence 

The Grading of Recommendations Assessment, Development, and Evaluation (GRADE) approach was used to evaluate the level of evidence [17]. The level of evidence was classified as high, moderate, low, or very low based on study limitations, indirectness of evidence, unexplained heterogeneity, imprecision of the results, and high probability of publication bias [18]. High-quality evidence was scored when all items were negative, moderate quality was scored when one item included serious risk, low quality if two items showed serious risk or one item showed very serious risk, or very low quality when three or more items had serious risk or two or more had very serious risk. This process was also performed by two authors, with the participation of a third one if disagreement occurred.

### 2.6. Data Synthesis and Analysis

Data analysis was performed with Review Manager statistical software (RevMan version 5.3). Data synthesis was presented by groups according to comparative groups as sham/control/placebo, manual therapy, or other physical therapy intervention and by follow-up as immediate (less than one week), short (1 to 12 weeks), and mid (12 to 24 weeks)-terms, since long-term (>24 weeks) data was not available. No other subgroup analysis was prespecified a priori.

Data extraction for the data analysis included sample size, means, and standard deviations of the outcomes. When the trial reported standard errors, they were converted to standard deviations. Mean and standard deviation were estimated from graphs when needed. If data were expressed as median and interquartile range, they were converted to mean and standard deviation as needed [19,20].

The between-groups mean difference (MD) with the 95% confidence interval (CI) was calculated for those outcomes assessed with the same instrument, e.g., pain intensity and pressure pain thresholds. Between-groups mean differences were converted to SMD when different instruments were used for the same outcome, e.g., pain-related disability. A random-effects model was used to determine the effect sizes (SMD). An effect size (SMD) of ≥0.8 was considered large, between 0.5 to 0.8 was considered moderate, and between 0.2 to 0.5 was considered small [21]. *p*-values < 0.05 were considered statistically significant. 

Cervical range of motion was pooled for each movement, i.e., flexion, extension, lateral-flexion, and rotation. When the trial calculated the total range of motion or either side separately for lateral-flexion and rotation, the mean was used in the analysis. If different groups received dry needling with different dosages, data were pooled in just one needling group for the meta-analyses. Finally, when two subgroups included the same intervention, e.g., dry needling, the sample size was adjusted by dividing the sample size as the Cochrane textbook recommends for avoiding duplication in the overall effect [22].

The I^2^ statistic was applied to determine the heterogeneity between the included trials. We used the interpretation of the Cochrane group as follows: 0–40% represented no relevant heterogeneity; 30–60% represented moderate heterogeneity, 50–90% suggested substantial heterogeneity, and 75–100% suggested considerable heterogeneity [22]. 

The asymmetry was evaluated using funnel plots in those analyses formed by at least five trials for indicating the possible risk of publication of small studies with negative results. Funnel plots of those analyses including more than 10 trials are presented as Supplementary Files. 

## 3. Results

### 3.1. Study Selection

**Figure 1:**
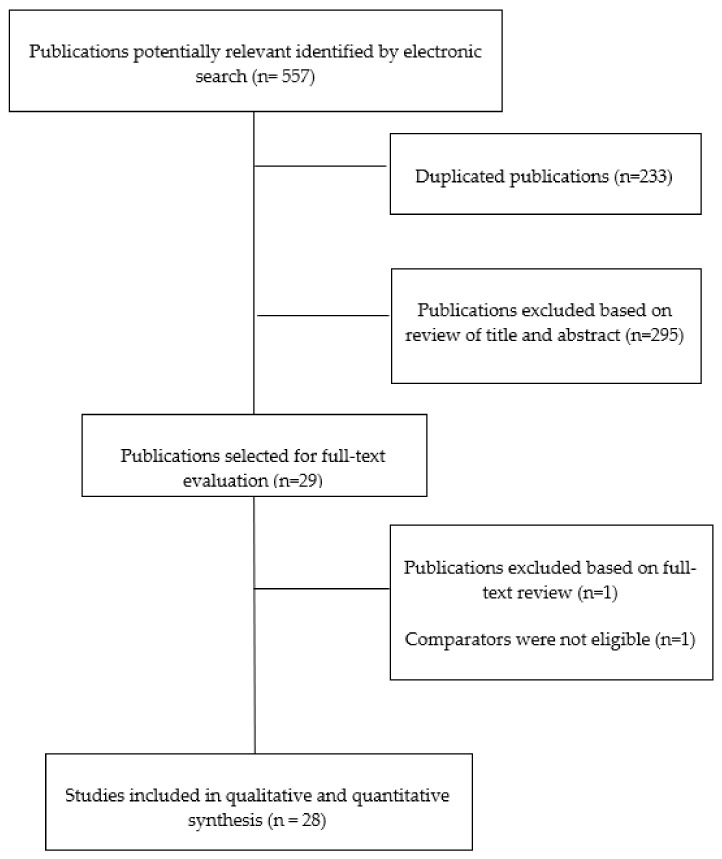
Preferred Reporting Items for Systematic Reviews and Meta-Analyses (PRISMA) flow diagram.

Fifty hundred and fifty-seven (*n* = 557) studies were initially identified. Three hundred and twenty-four (*n* = 324) studies remained after removing duplicates. Two hundred and ninety-five (*n* = 295) were excluded after the analysis of titles and/or abstracts, leaving 29 articles for final full-text review [23,24,25,26,27,28,29,30,31,32,33,34,35,36,37,38,39,40,41,42,43,44,45,46,47,48,49,50,51]. One article [34] was excluded because the comparator was acupuncture intervention and the placebo used laser. Finally, a total of 28 trials [25,26,27,28,29,30,31,32,33,35,36,37,38,39,40,41,42,43,44,45,46,47,48,49,50,51] were included in the meta-analysis (Figure 1).

### 3.2. Study Characteristics

Table 2 summarizes features of the participants on each trial. All studies targeted active TrPs (i.e., those that referred pain reproduced the patient’s symptoms) with the needle; seventeen (61%) targeted upper trapezius TrPs, seven (25%) targeted active TrPs in posterior cervical muscles, and the remaining four (14%) targeted just one muscle, e.g., levator scapulae, lower trapezius, anterior scalene, or sternocleidomastoid. Although all trials included one group receiving TrP dry needling, only 18 (65%) reported the presence of local twitch responses during the needling intervention. Fifty percent (*n* = 14) of the trials specified that needling intervention was applied by a physical therapist. There was heterogeneity in the comparative group, with seven trials comparing the application of dry needling against sham/control/no intervention, eight against manual therapy, and the remaining thirteen against other physiotherapy interventions ranging from high-power ultrasound to Kinesiotaping (see Table 1). All trials included pain intensity as the primary outcome, whereas twenty (72%) also assessed pain-related disability. Secondary outcomes were assessed in eighteen (pressure pain thresholds) and ten (cervical range of motion) trials. Dry needling interventions are described in Table 3.

### 3.3. Methodological Quality

The methodological quality total score ranged from 4 to 8 (mean: 6.6; SD: 1.15) from a total of 10 points. Twenty-three studies were of high methodological quality (≥6 points), and the remaining five were of low methodological quality (<6 points). No trial was able to blind therapists. The most frequent bias was blinding participants, since only seven trials were able to do so. The methodological score of each trial is shown in Table 4. 

### 3.4. Risk of Bias

**Figure 2:**
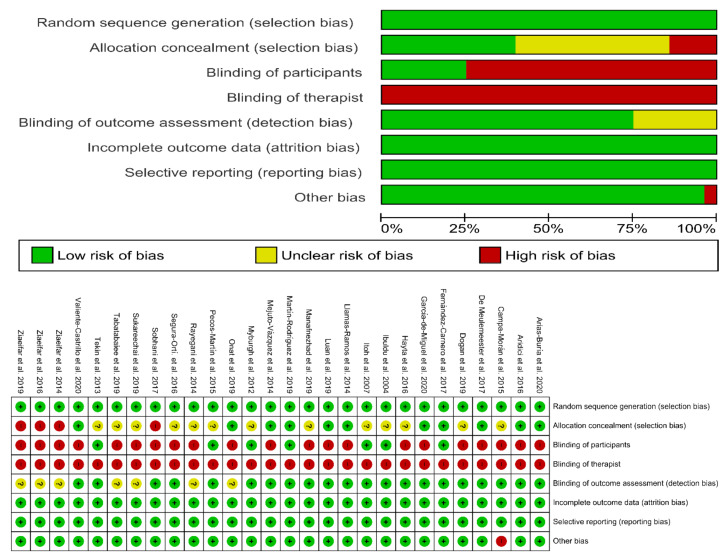
Plot of the risk of bias of the included studies.

Risk of bias assessment of the included trials is summarized in Figure 2. No trial was able to blind therapists, and twenty trials had high risks of bias for blinding participants. In general, the risk of bias of the included trials in the current meta-analysis was low.

### 3.5. Dry Needling and Neck Pain Intensity

**Figure 3:**
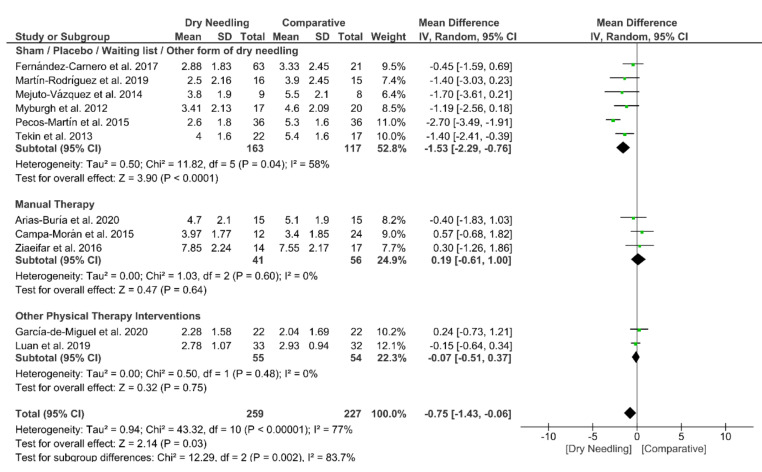
Mean differences (MD) comparing the immediate effects of dry needling alone against sham/placebo/waiting list/other forms of dry needling or manual therapy or other physical therapy interventions on pain intensity.

Dry needling exhibited a small overall significant effect (MD −0.75, 95% CI −1.43 to −0.06; *p* = 0.03 Z = 2.14, N = 486, *n* = 11 trials) for reducing neck pain immediately after the intervention vs. a comparison group but with substantial heterogeneity (I^2^ = 77%) between the trials (Figure 3). A significant effect (MD −1.53, 95% CI −2.29 to −0.76, *p* < 0.001) was found for the grouping analysis (*p* = 0.002) being significant comparing dry needling vs. sham/placebo/waiting list/other forms of dry needling (MD −1.53, 95% CI −2.29 to −0.76, *p* = 0.04). The funnel plot did not present potential publication bias (Figure S1).

**Figure 4:**
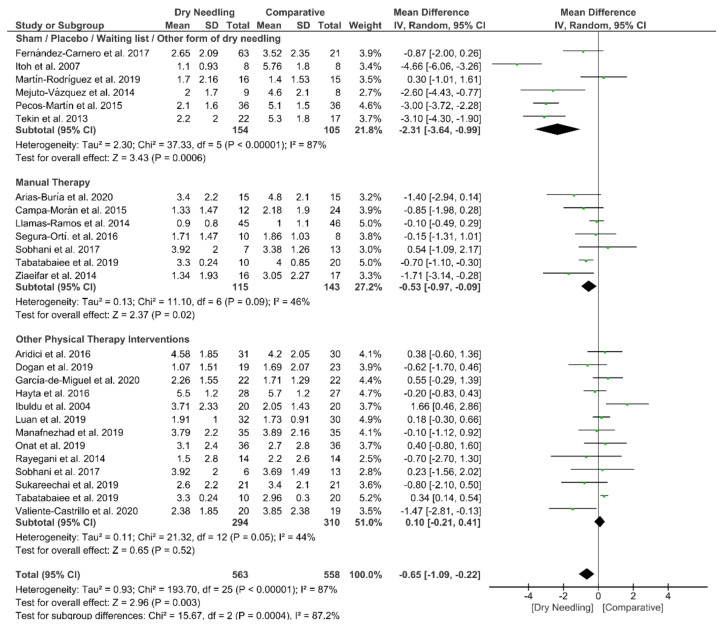
Mean differences (MD) comparing the short-term effects of dry needling alone against sham/placebo/waiting list/other forms of dry needling or manual therapy or other physical therapy. SD: standard deviation; CI: confidence interval.

Dry needling also showed a significant overall short-term effect (MD −0.65, 95% CI −1.09 to −0.22; *p* = 0.003, Z = 2.96, N = 1121, *n* = 24 trials) for reducing the intensity of neck pain as compared to a comparative group but, also, with considerable heterogeneity (I^2^ = 87%) between the trials (Figure 4). Significant subgroup differences (*p* = 0.0004, I^2^ = 87.2%) were observed when comparing dry needling with sham/placebo/waiting list/other forms of dry needling (MD −2.31, 95% CI −3.64 to −0.99, *p* < 0.001) and with manual therapy (MD −0.53, 95% CI −0.97 to −0.09, *p* = 0.02), but not when comparing with other physical therapy interventions (MD 0.10, 95% CI −0.21 to 0.41, *p* = 0.52). The funnel plot did not present a potential publication bias (Figure S2).

**Figure 5:**
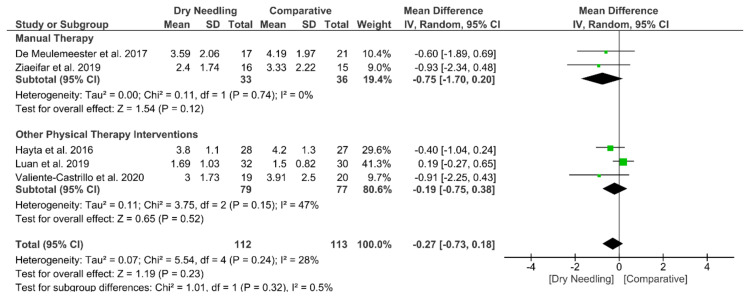
Mean differences (MD) comparing the mid-term effects of dry needling alone against sham/placebo/waiting list/other forms of dry needling or manual therapy or other physical therapy. SD: standard deviation; CI: confidence interval.

At mid-term, dry needling did not exhibit a significant overall effect (MD −0.27, 95% CI −0.73 to 0.18, *p* = 0.23, Z = 1.19, N = 225, *n* = 5 trials) for decreasing neck pain intensity when compared with a comparative group, with no significant heterogeneity (I^2^ = 28%) between the studies (Figure 5). No significant subgroup differences (*p* = 0.32, I^2^ = 0.5%) were observed. Table S1 summarizes the main results of the included studies.

### 3.6. Dry Needling and Pain-Related Disability

**Figure 6:**
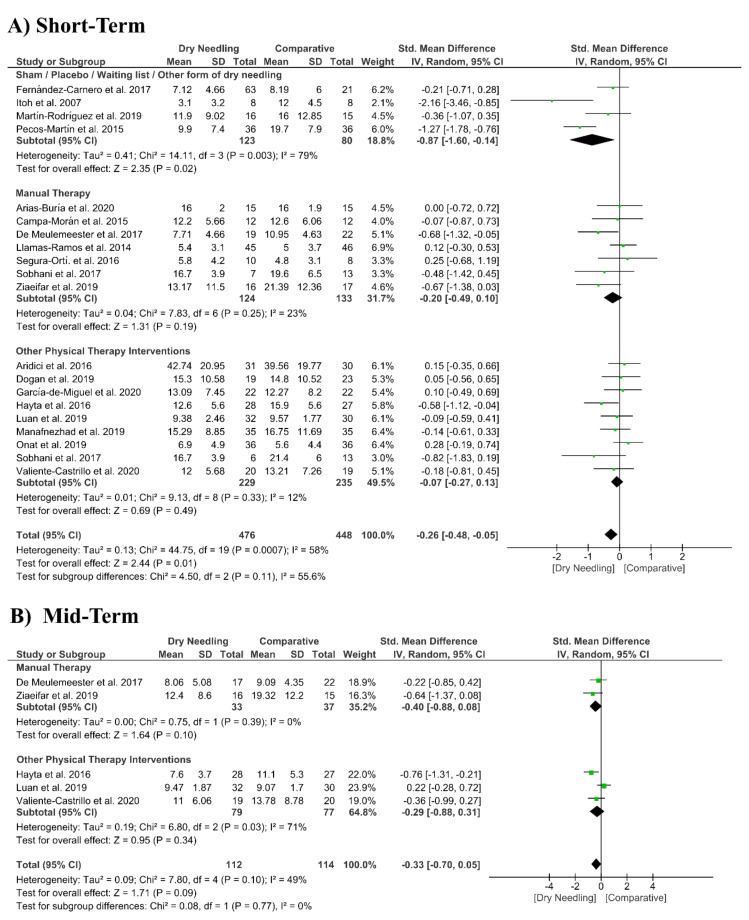
Standardized mean differences (SMD) comparing the effects of dry needling alone against sham/placebo/waiting list/other forms of dry needling or manual therapy or other physical therapy interventions on pain-related disability at the (**A**) short- and (**B**) mid-terms. SD:standard deviation; CI: confidence interval.

Dry needling had a significant overall small effect size (SMD −0.26, 95% CI −0.48 to −0.05, *p* = 0.001, Z = 2.44, N = 924, *n* = 20 trials) for improving pain-related disability at the short-term when compared with a comparative group but with moderate heterogeneity (I^2^ = 58%) among trials (Figure 6A). Significant differences were found when comparing dry needing with sham/placebo/waiting list/other forms of dry needling (SMD −0.87, 95% CI −1.60 to −0.14, *p* = 0.003) but not when compared with manual therapy (SMD −0.20, 95% CI −0.49 to 0.10, *p* = 0.19) or other physical therapy interventions (SMD −0.07, 95% CI −0.27 to 0.13, *p* = 0.49). The funnel plot presented asymmetry and publication bias (Supplementary Figure S3).

At mid-term follow-up, dry needling did not exhibit a significant overall effect (SMD −0.33, 95% CI −0.70 to 0.05, *p* = 0.09, Z = 1.71, N = 226, *n* = 5 trials) for reducing pain related-disability as compared to a comparative group, with moderate heterogeneity (I^2^ = 49%) among the trials (Figure 6B). No significant subgroup differences were found (*p* = 0.77, I^2^ = 0%). Table S1 summarizes the main results of the included studies.

### 3.7. Dry Needling and Pressure Pain Sensitivity (Pressure Pain Thresholds)

**Figure 7:**
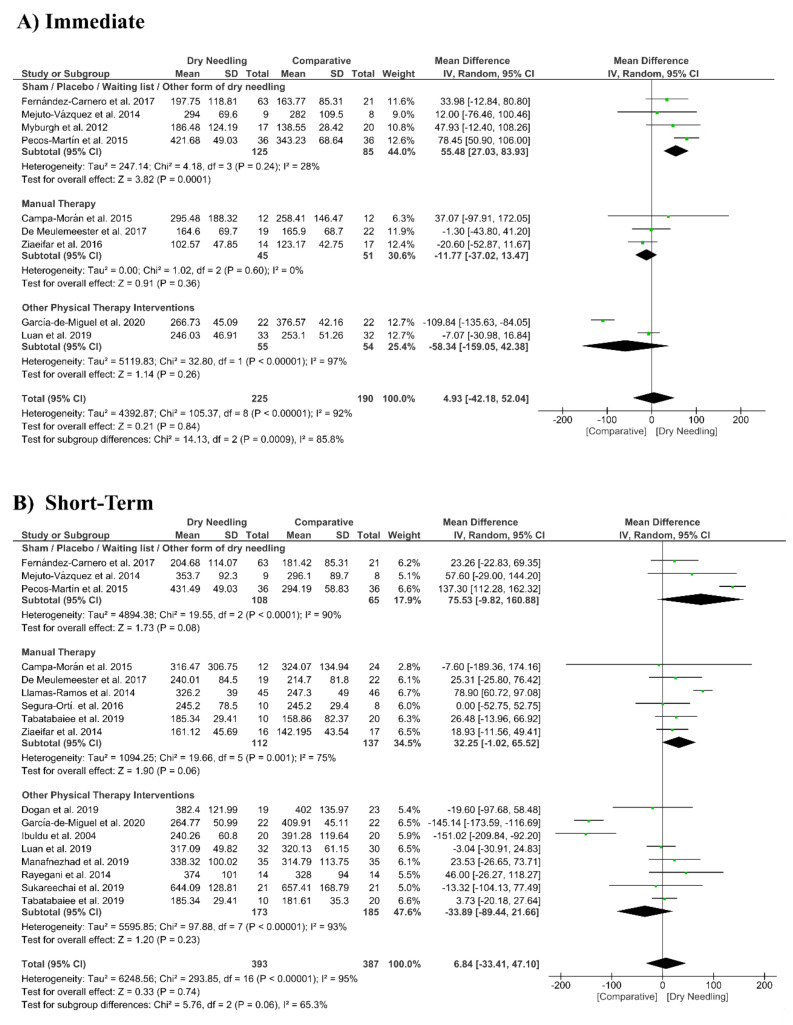
Mean differences (MD) comparing the effects of dry needling alone against sham/placebo/waiting list/other forms of dry needling or manual therapy or other physical therapy interventions on the pressure pain thresholds (kPa) (**A**) immediately after and (**B**) at the short-term. SD: standard deviation; CI: confidence interval.

Dry needling did not show a significant overall effect immediately after (MD 4.93 kPa, 95% CI −42.18 to 52.04, *n* = 415, Z = 0.21, *p* = 0.84, Figure 7A) and at short-term (MD 6.84 kPa, 95% CI −33.41 to 47.10, *n* = 780, Z = 0.33, *p* = 0.74, Figure 7B) for increasing the pressure pain thresholds vs. a comparative group. The funnel plot did not present a potential publication bias (Supplementary Figure S4).

The analysis also revealed considerable heterogeneity (I^2^ > 95%) between the studies. Only the subgroup comparing dry needling with sham/placebo/waiting list/other forms of dry needling had a significant immediate effect (MD 55.48 kPa, 95% CI 27.03 to 83.93, *p* < 0.001, Figure 7A).

### 3.8. Dry Needling and Cervical Range of Motion

**Figure 8:**
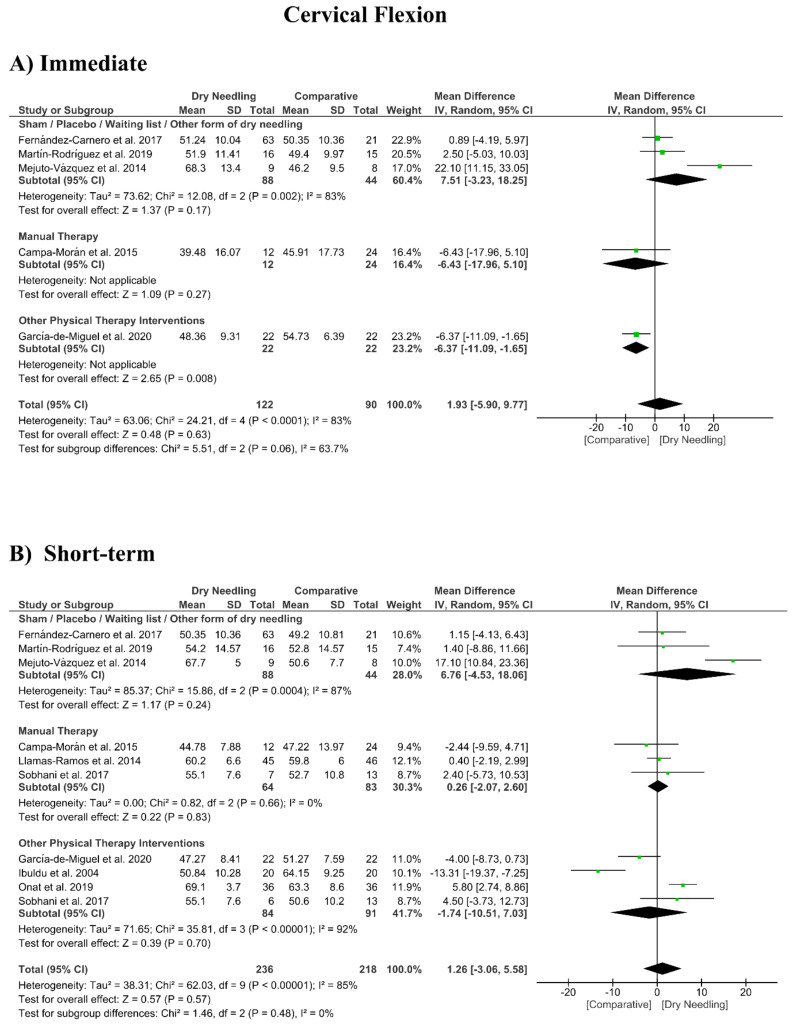
Mean differences (MD) comparing the effects of dry needling alone against sham/placebo/waiting list/other forms of dry needling or manual therapy or other physical therapy interventions on the cervical range of motion in flexion (**A**) immediately after and (**B**) at the short-term. SD: standard deviation; CI: confidence interval.

**Figure 9:**
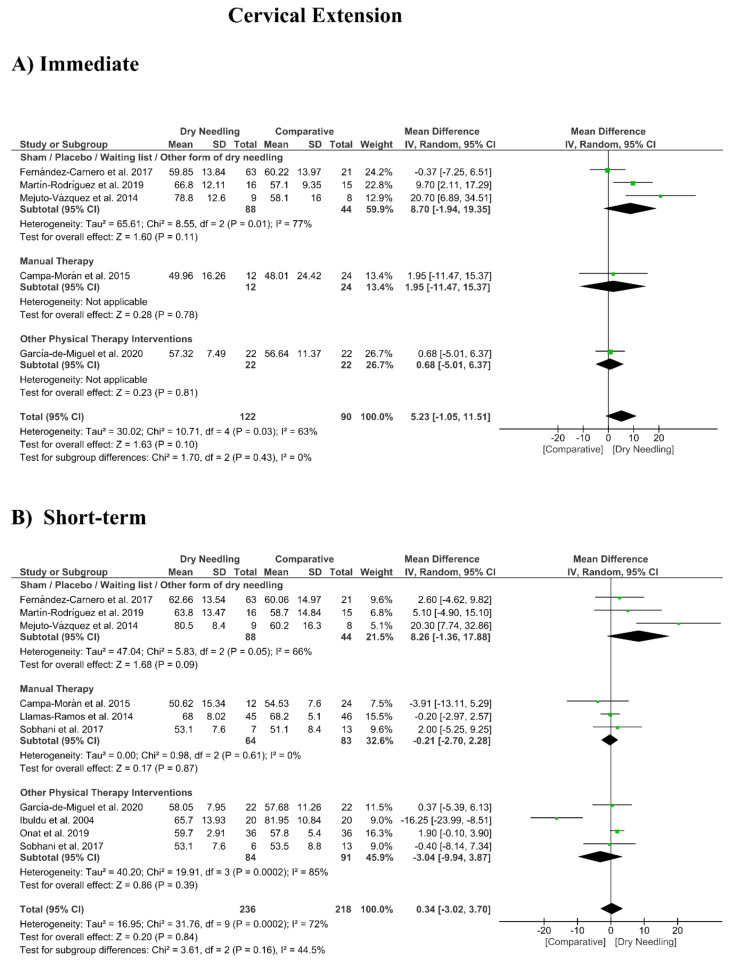
Mean differences (MD) comparing the effects of dry needling alone against sham/placebo/waiting list/other forms of dry needling or manual therapy or other physical therapy interventions on the cervical range of motion in extension (**A**) immediately after and (**B**) at the short-term. SD: standard deviation; CI: confidence interval.

**Figure 10:**
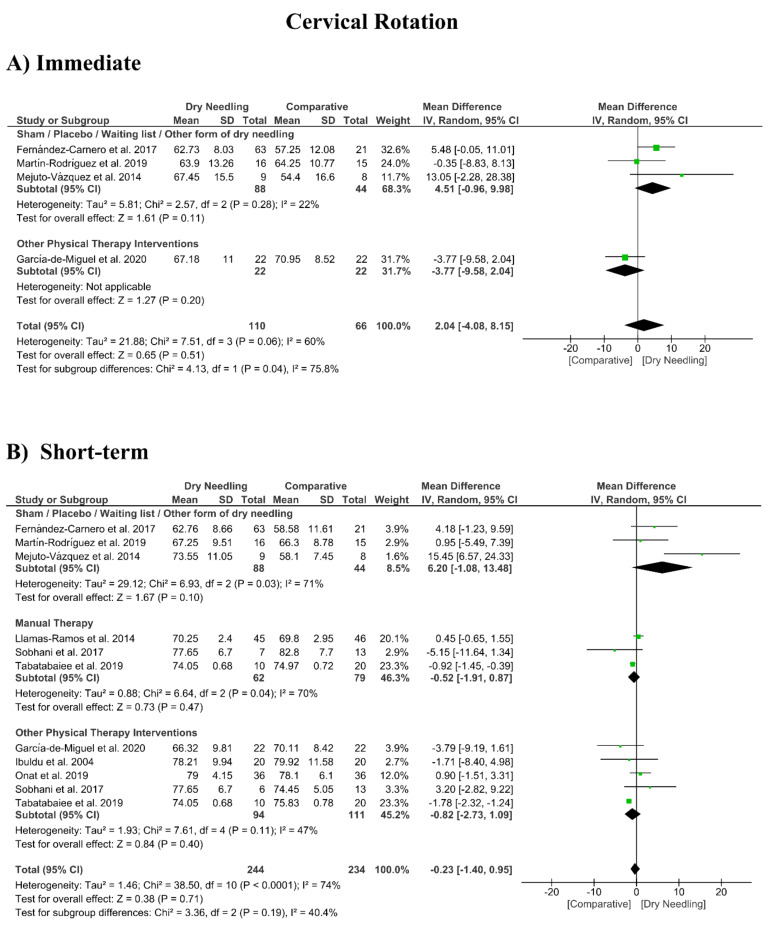
Mean differences (MD) comparing the effects of dry needling alone against sham/placebo/waiting list/other forms of dry needling or manual therapy or other physical therapy interventions on the cervical range of motion in rotation (**A**) immediately after and (**B**) at the short-term. SD: standard deviation; CI: confidence interval.

**Figure 11:**
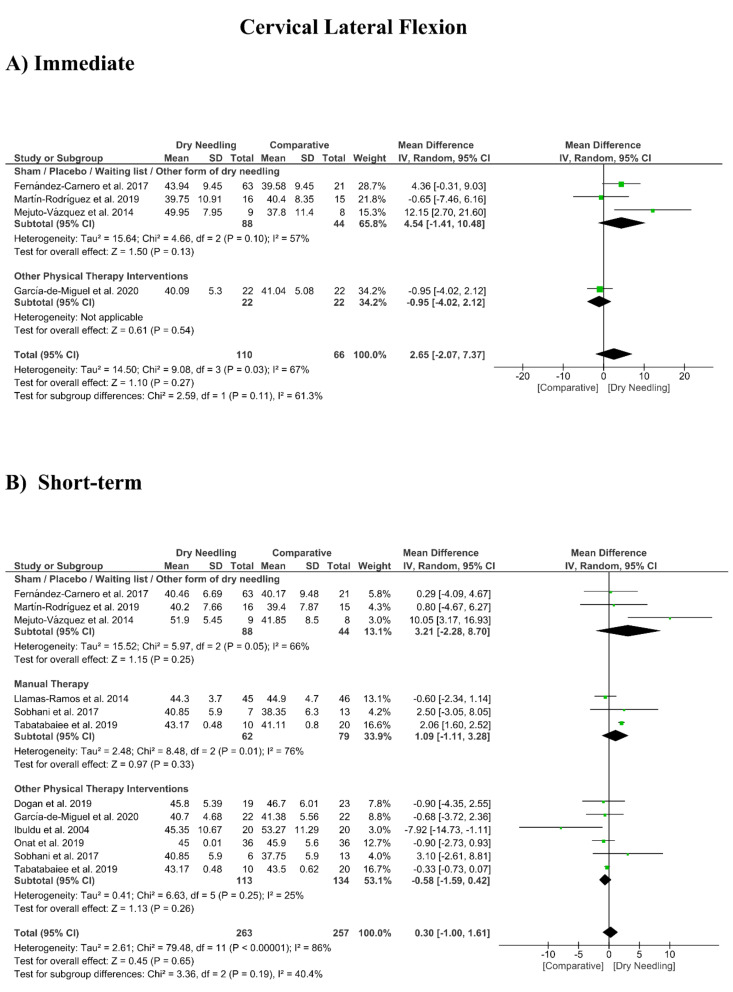
Mean differences (MD) comparing the effects of dry needling alone against sham/placebo/waiting list/other forms of dry needling or manual therapy or other physical therapy interventions on the cervical range of motion in lateral-flexion (**A**) immediately after and (**B**) at the short-term. SD: standard deviation; CI: confidence interval.

No significant overall effects of dry needling immediately after on the cervical range of motion when compared with a comparison group were observed: flexion (MD 1.93°, 95% CI −5.90° to 9.77°, *n* = 212, Z = 0.48, *p* = 0.63, Figure 8A), extension (MD 5.23°, 95% CI −1.05° to 11.51°, *n* = 212, Z = 1.63, *p* = 0.10, Figure 9A), rotation (MD 2.04°, 95% CI −4.08° to 8.15°, *n* = 176, Z = 0.65, *p* = 0.51, Figure 10A), and lateral-flexion (MD 2.65°, 95% CI −2.07° to 7.37°, *n* = 176, Z = 1.10, *p* = 0.27, Figure 11A). Similarly, no significant overall short-term effect of dry needling on cervical flexion (MD 1.26°, 95% CI −3.06° to 5.58°, *n* = 458, Z = 0.57, *p* = 0.57, Figure 8B), extension (MD 0.34°, 95% CI −3.02° to 3.70°, *n* = 454, Z = 0.20, *p* = 0.84, Figure 9B), rotation (MD −0.23°, 95% CI −1.40° to 0.95°, *n* = 478, Z = 0.38, *p* = 0.71, Figure 10B), and lateral-flexion (MD 0.30°, 95% CI −1.00° to 1.61°, *n* = 520, Z = 0.45, *p* = 0.65, Figure 11B) was found. All group analyses showed substantial heterogeneity. Table 3 summarizes the main results of the included studies. 

### 3.9. Adverse Events

Fifteen trials (53%, *n* = 15/28) reported information about adverse effects, with all of them reporting just minor events, and none reported any serious adverse effects [27,28,29,30,31,32,33,38,39,40,41,43,45,50]. Post-needling soreness was the most common adverse event and was reported in 53% (8/15) of the trials [27,28,32,38,40,43,45,48] and resolved spontaneously in 24–48h without further treatment. Thirteen (47%, *n* = 13/28) of the included studies [23,24,25,30,35,36,42,44,46,47,48,49,51] did not report any information about adverse events (Table 5).

### 3.10. Quality of Evidence (GRADE)

Table 6 summarizes the RoB, inconsistency of the results, indirectness of evidence, imprecision of results, and high probability of publication bias for determining the level of evidence according to GRADE assessment. The serious/very serious inconsistency of the results (heterogeneity) and the serious/very serious impression downgraded the evidence level of dry needling to low or moderate.

## 4. Discussion

### 4.1. Trigger Point Dry Needling and Neck Pain

This meta-analysis aimed to compare the effects of dry needling alone against any comparative group, e.g., sham, control, no intervention, or other physical therapy interventions applied over TrPs associated with neck pain symptoms. We found moderate-to-low evidence supporting the effectiveness of dry needling for improving pain intensity and related-disability as compared with a comparative group immediately after and at short-, but not at mid-, term follow-ups. The effects were observed when dry needling was compared with sham, placebo, or a waiting list. No significant effect on pressure pain sensitivity or cervical range of motion was found. The RoB of the included trials was relatively low, but the inconsistency (heterogeneity) or the imprecision of the results downgraded the evidence level according to the GRADE.

This is an updated meta-analysis analyzing the effectiveness of the application of dry needling alone on the pain intensity, related-disability, pressure pain sensitivity, and cervical range of motion in patients with myofascial TrPs associated with neck pain symptoms. Liu et al. [13] concluded that dry needling was effective immediately after (SMD −1.91, 95% CI −3.10 to −0.73) and at four weeks (SMD −1.07, 95% CI −1.87 −0.27) when compared with the control or sham. The current updated meta-analysis also observed that dry needling was more effective than sham/placebo/waiting list/other forms of dry needling immediately after (MD −1.53, 95% CI −2.29 to −0.76) and at short-term (MD −2.31 points, 95% CI −3.64 to −0.99). 

We also found low-quality evidence supporting a small positive overall effect (SMD −0.26, 95% CI −0.48 to −0.05) of dry needling for improving related disability when compared with a comparison group at the short-term. The effects were only observed comparing dry needling against sham/placebo/waiting list/other forms of dry needling. Based on the current evidence, it seems that the application alone of dry needling targeting active TrP may be effective for the treatment of neck pain (low-to-moderate evidence); however, the effects were mostly observed at the short-term (2–12 weeks after treatment) and vs. sham/placebo/waiting list/other forms of dry needling but not against manual therapy or physical therapy interventions. In fact, the topic of a proper sham needling approach is questioned, since sham needling interventions used in the current literature are highly diverse, limiting the comparability of blinding effectiveness across current studies [52]. It has been supported that sham needling could also have a potential therapeutic effect, probably related to cognitive factors, such as expectative or placebo [52].

It is important to consider if the observed changes on pain intensity were clinically relevant. We reported an overall mean decrease of pain intensity of −0.75 points (95% CI −1.43 to −0.06) immediately after and of −0.65 points, 95% CI −1.09 to −0.22 at the short-term after applying dry needling alone. These between-groups mean differences did not reach the minimal clinically important difference (MCID) of 2.1 points specifically described for patients with mechanical neck pain [53] or the general MCID of 1.4 points determined by Bijur et al. [54]. Nevertheless, comparing dry needling vs. sham/placebo/waiting list, changes observed immediately after (−1.53 points, 95% CI −2.29 to −0.76) and at the short-term (−2.31 points, 95% CI −3.64 to −0.99) were slightly superior to the MCID reported by Bijur et al. [54] and Cleland et al. [53], respectively. Nevertheless, the lower-bound estimate of the confidence intervals did not surpass the MCID.

We did not find significant differences for the application of dry needling or other interventions on the pressure pain sensitivity and cervical range of motion. The results suggest that dry needling has similar effects on these outcomes than manual therapy or other physical therapy interventions, although this conclusion should be considered with caution (very low evidence). Current results would agree with recent theories supporting a common neurophysiological mechanism for manual therapy [55] or needling approaches [56], explaining the hypoalgesic effects and improvements in range of motion observed. In such a scenario, clinicians could choose the application of an intervention according to the individual clinical presentation of each patient based on his/her beliefs, preferences, or expectative.

Although our meta-analysis could be considered an updated version of the Liu et al. [13] paper, several differences can be observed: (1) Liu et al. [13] included trials analyzing wet needling, whereas we included only dry needling; (2) Liu et al. [13] only included pain intensity as the outcome in their quantitative analysis, whereas our study included other outcomes such as related-disability, pressure pain sensitivity, and neck range of motion; (3) Liu et al. [13] considered 9–28 days after the intervention as a mid-term follow-up period, when it is more appropriate to be considered as a short-term; and (4) Liu et al. [13] included trials conducted on post-stroke patients presenting with shoulder pain [57], whereas we included patients with neck pain of musculoskeletal origin associated to TrPs. Therefore, it seems that this meta-analysis represents the most updated information about the effects of dry needling on patients with TrPs associated with neck pain of musculoskeletal origin. 

### 4.2. Adverse Events Associated to Trigger Point Dry Needling

The safety of dry needling is under debate in the current literature due to the presence of potential adverse events. Carlesso et al. [58] defined an adverse event “as a sequela of medium-term duration with any symptom perceived as unacceptable to the patient and requiring further treatment”.

Two previous studies investigating the presence of adverse events after the application of dry needling reported that bleeding (16%), bruising (7.7%), and pain during/after treatment (5.9%) were the most prevalent adverse events [59,60]. All these events were considered as minor [59,60]. Fifty percent of the trials included in our meta-analysis reported the presence of post-needling soreness as the main minor adverse event, supporting that dry needling is a potentially safe intervention. However, major adverse events, e.g., pneumothorax, have been also reported in some cases, although their rate is less than 0.1% (1 per 1024 needling treatments) and depend on the anatomical location. In fact, case reports describing pneumothorax after dry needling have applied the intervention over the thoracic, and not cervical spine, muscles [61,62]. Although dry needling could be considered a safe treatment if properly applied, potential risks associated with its application on each body area where it is applied should be taken into account. In fact, recent studies have proposed different positions [63] or the use of echography [64] for improving the safety of dry needling application.

### 4.3. Strengths and Limitations

The results of this meta-analysis should be considered according to its potential strengths and limitations. Potential strengths include the comprehensive literature search, rigorous statistical analysis, and the inclusion of randomized controlled trials of high methodological quality. Among the limitations, first, dry needling interventions were highly heterogeneous in the number of sessions, the frequency of application, presence or absence of local twitch responses, or musculature receiving the treatment. In addition, it should be noted that current results come from including all dry needling protocols in the same group, i.e., we compared the application of dry needling for 10 min or 90 s during a single session or different sessions with heterogeneous protocols of manual therapy or other physiotherapy interventions (e.g., 10 sessions over four weeks). Second, the heterogeneity and imprecision of the results of the trials were serious; therefore, the results should be considered with caution at this stage. Nevertheless, this heterogeneity led to the use of a random-effects model rather than the use of a fixed-effects model [65]. Third, the number of trials analyzing mid-term effects was small (*n* = 3), and no long-term data were available. Therefore, a greater number of high-quality clinical trials investigating mid- and long-term effects of dry needling could lead to different results. 

### 4.4. Clinical and Research Implications

Considering that this is the most updated meta-analysis evaluating the effectiveness of applying dry needling in isolation in patients with neck pain associated to muscle TrPs, several questions need to be elucidated in future trials. First, most studies investigated immediate or short-term effects, with just a small number of studies investigating mid- and long-term follow-ups. Second, trials in this meta-analysis investigated the isolated application of dry needling without any other intervention, which does not represent common clinical practice. 

Future high-quality clinical trials examining the long-term effects of the inclusion of dry needling into multimodal physical therapy programs is more effective than not including them. Additionally, since neck pain is characterized by motor control changes, it would be interesting to investigate if the inclusion of dry needling could lead to changes in muscle strength outcomes. In fact, a recent meta-analysis reported medium effect sizes for dry needling to enhance the force production in those with neck pain (moderate evidence), although this analysis was based on just two studies [66]. 

Finally, it should be noted that only 50% (*n* = 14) of the trials included in this study specified that the dry needling intervention was applied by a physical therapist. This would be a relevant topic to research, since the clinical reasoning behind the application of needling interventions, e.g., traditional Chinese medicine vs. Western occidental reasoning, may potentially modify the procedure and the outcomes. In fact, the meta-analysis by Gattie et al. [67] investigated the effects of dry needling applied just by physical therapists, although further research is clearly needed.

## 5. Conclusions

This systematic review and meta-analysis found moderate-to-low evidence suggesting that dry needling can be effective for improving neck pain intensity and related disability when compared with a comparative group immediately after and at short-, but not at mid-, term follow-ups in people with myofascial TrPs associated with neck pain symptoms. The effects were mostly observed when dry needling was compared with sham/placebo/waiting list/other forms of dry needling but not against other physical therapy interventions. No significant effects on the pressure pain sensitivity or cervical range of motion were found. The RoB of the clinical trials included was relatively low, but the inconsistency (heterogeneity) and imprecision of the results downgraded the level of evidence. 

## Figures and Tables

**Table 1:** Database formulas during the literature search.

**PubMed Search Formula**
#1 “Dry Needling” (Mesh) OR “Trigger Point Acupuncture” (Title/Abstract) OR “Needling Therapy” (Title/Abstract) OR “Intramuscular Stimulation” (Title/Abstract)
#2 “Placebos” (Mesh) OR “Control Groups” (Mesh) OR “Physical Therapy Modalities” (Mesh)
OR “Cervical Pain” (Title/Abstract) OR “Mechanical Neck Pain” (Title/Abstract) OR “Myofascial Neck Pain” (Title/Abstract)
#4 #1 AND #2 AND #3
**CINAHL/Medline (via EBSCO) Search Formula**
#1 “Dry Needling” OR “Trigger Point Acupuncture” OR “Needling Therapy” OR “Intramuscular Stimulation”
#2 “Placebos” OR “Control Groups” OR “Physical Therapy Modalities”
#3 “Neck Pain” OR “Non-Specific Neck Pain” OR “Cervicalgia” OR “Cervical Pain” OR “Mechanical Neck Pain” OR “Myofascial Neck Pain”
#4 #1 AND #2 AND #3
**SCOPUS Search Formula**
TITLE-ABS-KEY (“Dry Needling” OR “Trigger Point Acupuncture” OR “Needling Therapy” OR “Intramuscular Stimulation”) AND TITLE-ABS-KEY (“Placebos” OR “Control Groups” OR “Physical Therapy Modalities”) AND TITLE-ABS-KEY (“Neck Pain” OR “Non-Specific Neck Pain” OR “Cervicalgia” OR “Cervical Pain” OR “Mechanical Neck Pain” OR “Myofascial Neck Pain”)
**PEDro Search Formula**
Abstract & Title: Neck Pain, Myofascial Pain Syndrome
Therapy: Dry Needling
Method: Clinical trial
When Searching: AND
**WOS Search Formula**
(“Dry Needling” OR “Trigger Point Acupuncture” OR “Needling Therapy” OR “Intramuscular Stimulation”) AND (“Placebos” OR “Control Groups” OR “Physical Therapy Modalities”) AND (“Neck Pain” OR “Non-Specific Neck Pain” OR “Cervicalgia” OR “Cervical Pain” OR “Mechanical Neck Pain” OR “Myofascial Neck Pain”)
**Cochrane Library Search Formula**
#1 Mesh: Dry Needling
#2 Mesh: Placebos
#3 Mesh: Neck Pain
#4 Trigger Point Acupuncture
#5 Needling Therapy
#6 Intramuscular Stimulation
#7 Mesh: Control Groups
#8 Mesh: Physical Therapy Modalities
#9 Nonspecific Neck Pain
#10 Cervicalgia
#11 Cervical Pain
#12 Mechanical Neck Pain
#13 Myofascial Neck Pain
#14 #1 OR #4 OR #5 OR #6
#15 #2 OR #7 OR #8
#16 #3 OR #9 OR #10 OR #11 OR #12 OR #13
#17 #14 AND #15 AND #16

**Table 2:** Characteristics of the samples in each included trials.

Study	Diagnosis	Group	Total (Male/Female)	Age (SD), y	Pain Duration
Ibuldu et al. 2004 [36]	Myofascial Pain Syndrome	G1: DN + Self-Stretching	20	35.3 (9.2)	38.5 (31.95) m
		G2: Laser + Self-Stretching	20	33.9 (10.35)	32.95 (28.6) m
		G3: Placebo laser + Self-Stretching	20	32.35 (6.9)	36.95 (33.65) m
Itoh et al. 2007 [33]	Chronic Neck Pain	G1: TrP-DN	8	62.3 (10.1)	2.9 (2.7) y
		G2: Non-TrP-DN	8	65.0 (10.5)	3.3 (3.9) y
		G3: Sham Acupuncture	7	65.0 (10.5)	2.3 (1.5) y
		G4: Acupuncture	8	62.3 (11.0)	3.2 (3.2) y
Myburgh et al. 2012 [27]	Myofascial Pain Syndrome	G1: TrP-DN	17	46.1	NR
		G2: TrP-SDN	20	46.1	NR
Tekin et al. 2012 [46]	Myofascial Pain Syndrome	G1: TrP-DN	22 (5/17)	42.9 (10.9)	63.5 (50.7) m
		G2: TrP-Sham DN	17 (3/14)	42.0 (12.0)	57.9 (48.3) m
Llamas-Ramos et al. 2014 [32]	Mechanical Neck Pain	G1: TrP-DN	47 (17/30)	31 (3)	7.4 (2.6) m
		G2: TrP-MT	47 (15/32)	31 (2)	7.1 (2.9) m
Ziaeifar et al. 2014 [35]	Myofascial Pain Syndrome	G1: TrP-DN	16	30.05 (9.9)	NR
		G2: TrP-MT	17	26.5 (8.6)	NR
Mejuto-Vázquez et al. 2014 [28]	Acute Mechanical Neck Pain	G1: TrP-DN	9 (4/5)	24 (7)	3.4 (0.7) d
		G2: No intervention	8 (4/4)	25 (4)	3.1 (0.8) d
Rayegani et al. 2014 [51]	Myofascial Pain Syndrome	G1: TrP-DN	14	32 (10) 38.6	9.6 (8.4) m 9.8 (9.6) m
		G2: Physical Therapy	14	(4.2)	
Campa-Mran et al. 2015 [41]	Myofascial Chronic Neck Pain	G1: TrP-DN + Passive Stretching	12 (3/9)	53.9 (12.7)	10.0 (2.9) m
		G2: Soft tissue techniques	12 (2/10)	45.8 (15.4)	11.8 (4.4) m
		G3: MT	12 (2/10)	48.7 (10.2)	14.0 (3.6) m
Pecos-Martín et al. 2015 [25]	Chronic Mechanical Neck Pain	G1: TrP-DN	36 (6/30)	23 (5)	5.7 (2.6) m
		G2: Non-TrP-DN (Sham)	36 (8/28)	23 (6)	7 (2.8) m
Aridici et al. 2016 [42]	Myofascial Pain Syndrome	G1: TrP-DN	31 (5/26)	40.5 (10.1)	7.5 (3.0)
		G2: High power pain threshold ultrasound therapy	30 (3/27)	38.1 (11.4)	7.75 (3.0)
Segura-Ortí et al. 2016 [50]	Myofascial Pain Syndrome	G1: TrP-DN	12 (4/8)	30.0 (9.5)	NR
		G2: Strain Counter-strain Technique	10 (3/7)	34.1 (11.5)	NR
		G3: Sham Strain Counter-strain	12 (2/10)	32.9 (9.5)	NR
Hayta et al. 2016 [37]	Myofascial Pain Syndrome	G1: TrP-DN	28 (7/21)	NR	NR
		G2: Kinesiotaping	27 (3/24)	NR	NR
Ziaeifar et al. 2016 [23]	Myofascial Pain Syndrome	G1: TrP-DN	14 (0/14)	30.1 (10.4)	NR
		G2: TrP-MT	17 (0/17)	26.6 (9.4)	NR
Fernández-Carnero et al. 2017 [38]	Cervical Myofascial Pain	G1: 4 LTR DN	21 (7/14)	29.7 (11.9)	9.7 (17.0) m
		G2: 6 LTR DN	21 (5/16)	24.25 (9.4)	16.85 (38.5) m
		G3: +6 LTR DN	21 (5/16)	26.45 (10.7)	19.2 (22.15) m
		G4: Non-TrP DN	21 (4/17)	28.2 (11.4)	8.4 (15.4) m
De Meulemeester et al. 2017 [40]	Myofascial Neck/Shoulder Syndrome	G1: TrP-DN	22	40.5 (8.3)	3: <3m; 19: >3m:
		G2: TrP-MT	0	36.1 (10.7)	4: <3m; 16: >3m
Sobhani et al. 2017 [49]	Chronic Mechanical Neck Pain	G1: DN + Passive stretching	13	34.6 (10.5)	12.6 (4.4) m
		G2: MT	13	35.9 (11.4)	15.1 (7.5) m
		G3: Kinesiotaping	13	34.6 (9.1)	16.1 (7.6) m
Luan et al. 2019 [31]	Myofascial Pain Syndrome	G1: DN	32 (11/21)	33.1 (12.8)	8.3 (3.1) m
		G2: Extracorporeal Shock Wave	30 (8/22)	32.5 (10.6)	8.9 (2.7) m
Dogan et al. 2019 [39]	Myofascial Pain Syndrome	G1: DN	19	32.4 (12.4)	12 (4–48) m
		G2: Kinesiotaping	23	33.6 (9.1)	12 (4–60) m
Manafnezhad et al. 2019 [30]	Non-Specific Neck Pain	G1: DN	35	39.2 (7.2)	12 (3–60) m
		G2: Extracorporeal Shock Wave	35	37 (9.1)	12 (3–80) m
Martín-Rodríguez et al. 2019 [29]	Non-Specific Neck Pain	G1: TrP-DN	17 (6/11)	43.6 (12.1)	88.5 (105.1) m
		G2: Non-TrP- DN	14 (4/13)	42.5 (12.3)	58.9 (48.5) m
Tabatabaiee et al. 2019 [47]	Myofascial Pain Syndrome	G1: Latent-TrP DN	20	23.6 (1.8)	NR
		G2: TrP-MT	20	23.5 (1.6)	NR
		G3: Phonophoresis with betamethasone	20	23.9 (3.1)	NR
Onat et al. 2019 [26]	Neck Pain	G1: TrP-DN + Home Exercise Program	36 (7/29)	44.1 (14.2)	NR
		G2: Kinesiotaping + Home Exercise Program	36 (10/26)	45.1 (12.5)	NR
Ziaeifar et al. 2019 [24]	Myofascial Pain Syndrome	G1: TrP-DN	16	30.05 (9.9)	NR
		G2: TrP-MT	17	26.5 (8.6)	NR
Sukareechai et al. 2019 [48]	Myofascial Pain Syndrome	G1: TrP-DN	21 (0/21)	42.7 (12.4)	36 (3, 120) m
		G2: Radial Shockwave	21 (2/19)	38.2 (11.9)	24 (1, 120) m
Arias-Buría et al. 2020 [43]	Mechanical Neck Pain	G1: TrP-DN	15 (10/5)	21 (3)	7.5 (1.3) m
		G2: TrP-MT	15 (11/4)	22 (2)	8.0 (1.1) m
Valiente-Castrillo et al. 2020 [45]	Chronic Myofascial Neck Pain	G1: TrP-DN	20 (4/16)	40.3 (11.95)	43.4 (56.55) m
		G2: TrP-DN + pain neuroscience education	21 (2/19)	40.35 (8.0)	64.95 (62.9) m
		G3: Usual Care	19 (3/16)	42.35 (9.4)	56.3 (67.75) m
García-de-Miguel et al. 2020 [44]	Unilateral Mechanical Neck Pain	G1: TrP-DN	22 (9/13)	25.45 (8.5)	>3 m
		G2: PENS	22 (7/15)	24.15 (9.4)	>3 m

TrP: trigger point, DN: dry needling, SDN: superficial dry needling, G: group, MT: manual therapy, m: months, y: years, d: days, and NR: not reported. PENS: Percutaneous Nerve Electrical Stimulation.

**Table 3:** Characteristics of the dry needling intervention of the included studies.

Study	Group	TrP criteria	Technique Used	No. Punctures for Patient in Every Intervention	Needle Approach (Targeted Muscles or Tendon)	Gauge (mm)	Depth (mm)	Time of DN	Frequency of Incisions (Hz)	Number of Incisions in Every Needle Intervention	LTR	Therapist that Performed Intervention
Ibuldu et al. 2004 [36]	G1: DN	Yes	NR	1	Upper trapezius	0.25 × 25	NR	NR	NR	NR	NR	Physician
Itoh et al. 2007 [33]	G1: DN-Trp	Yes	“sparrow pecking” technique	NR	Splenius capitis, Upper trapezius, sternocleidomastoid, scalenus, levator scapulae, suboccipital	0.2 × 0.50 mm	20 mm	10 min	1	The manipulation was stopped when the LTR was elicited	Yes	Acupuncturist
	G2: Acupuncture	No	“sparrow pecking” technique	9	GB20, GB21, BL10, BL11, S12, S13, TE5, LI4, SI3	0.2 × 0.40 mm	20 mm	10min	1	When the subject felt dull pain or the acupuncture sensation (de qi), the manipulation was stopped	No	Acupuncturist
	G3: DN-Non-TrP	Yes	“sparrow pecking” technique	NR	Splenius capitis, upper trapezius, sternocleidomastoid, scalenus, levator scapulae, suboccipital	0.2 × 0.40 mm	0 mm	10 min	1	The manipulation was stopped when the LTR was elicited	Yes	Acupuncturis
Myburgh et al. 2012 [27]	G1: DN	Yes	Repeated fanning needling insertion	1	Upper trapezius	32 × 0.25 mm	No less than 10 mm	90 sg	NR	Elicit and exhaust LTR	Yes	Clinician
	G2: Superficial DN	Yes	The needle inserted into the epidermis until	1	Upper trapezius	32 × 0.25 mm	5 mm	90 sg	1	1	No	Clinician
Tekin et al. 2012 [46]	G1: DN	Yes	Needle moved forward until the TrP was reached	6	Neck and shoulder muscles	0.25 × 0.25 mm	Until muscle	NR	1	1	No	Physician
	G2: Sham-DN	Yes	The blunted needle for sham dry needling	6	Neck and shoulder muscles	0.25 × 0.25 mm	Until skin	NR	1	1	No	Physician
Llamas-Ramos et al. 2014 [32]	G1: DN	Yes	Hong	1	Upper trapezius	0.30 × 30 mm	10–15 mm	20–30 sg	1	Once the first LTR was obtained, the needle was moved up and down	Yes	Physiotherapist
Mejuto-Vázquez et al. 2014 [28]	G1: DN	Yes	Hong	1	Upper trapezius	0.30 × 30 mm	10–15 mm	20–30 sg	1	Once the first LTR was obtained, the needle was moved up and down	Yes	Physiotherapist
Ziaeifar et al. 2014 [35]	G1: DN	Yes	Hong	1	Upper trapezius	NR	NR	NR	NR	Was repeatedly needled forward and backward to the TrP until there were no more LTRs	Yes	Physiotherapist
Rayegani et al. 2014 [51]	G1: DN	Yes	NR	2	Upper trapezius	23-gauge needle	NR	NR	NR	NR	No	Physician
Campa-Moran et al. 2015 [41]	G1: DN	Yes	Hong	2	Levator scapulae and upper trapezius muscles	0.25 × 25 mm	Until muscle	2 min	At least 3 times at each point	The needle insertions were repeated to achieve at least three LTR	Yes	Physiotherapist
Pecos-Martín et al. 2015 [25]	G1: TrP-DN	Yes	Hong	1	Lower trapezius	0.25 × 25 mm	Until muscle	NR	NR	8-10 times	No	Physiotherapist
	G2: Non-TrP-DN	No	Hong	1	Lower trapezius	0.25 × 25 mm	1.5cm medially from TrP	NR	NR	8–10 times	No	Physiotherapist
Aridici et al. 2016 [42]	G1: DN	Yes	Hong	1	Upper trapezius	22-gauge needle and 1.5 inch	Until muscle	NR	NR	8–10 times	Yes	Physician
Hayta et al. 2016 [37]	G1: DN	Yes	Manual stimulation was produced (at the TrP) by rotating the needle counterclockwise	3	Trapezius	0.25 × 25 mm	Inside of muscle	10–20 min	1	1	No	NR
Segura-Ortí et al. 2016 [50]	G1: DN	Yes	Hong	1	Upper trapezius	0.25 × 25 mm	Inside of muscle	NR	NR	Needling at the TrP was continued until the LTR was exhausted	Yes	Physiotherapist
Ziaeifar et al. 2016 [23]	G1: DN	Yes	Hong	1	Upper trapezius	0.30 × 50 mm	Inside of muscle	NR	NR	The procedure was repeated until there was no more LTR	Yes	Therapist
Fernández-Carnero et al. 2017 [38]	G1: No-LTR-DN	Yes	Hong	1	Upper trapezius	0.32 × 40 mm	Inside of muscle, 1.5 cm away from TrP	NR	NR	1	No	Therapist
	G2: 4-LTR-DN	Ye	Hong	1	Upper trapezius	0.32 × 40 mm	Inside TrP	NR	NR	10 times	Yes	Therapist
	G3: 6-LTr-DN	Ye	Hong	1	Upper trapezius	0.32 × 40 mm	Inside TrP	NR	NR	10 times	Yes	Therapist
	G4: More-6-LTR-DN	Ye	Hong	1	Upper trapezius	0.32 × 40 mm	Inside TrP	NR	NR	10 times	Yes	Therapist
Sobhani et al. 2017 [49]	G1: DN	Yes	NR	2	Upper trapezius and levator scapulae muscles	NR	NR	NR	20 min	NR	No	Therapist
Dogan et al. 2019 [39]	G1: DN	Yes	Hong and the needles were kept in the TrP for ten minutes, after which they were turned counterclockwise several times	1	Upper trapezius	0.20 × 40 mm	Until TrP	10 min	NR	At least 3 insertions and 1 LTR	Yes	Physician
Luan et al. 2019 [31]	G1: DN	Yes	Hong	1	Upper trapezius	0.30 × 50 mm	30–35 mm	NR	NR	10	Yes	Physiotherapist
Manafnezhad et al. 2019 [30]	G1: DN	Yes	Hong	1	Upper trapezius	0.30 × 0.50 mm	Until TrP	1–2 min	NR	Until at least one or two LTR were obtained	Yes	Physiotherapist
Martín-Rodríguez et al. 2019 [29]	G1: DN	Yes	Hong	1	Sternocleidomastoid muscle	0.25 × 0.25 mm	Until TrP	NR	NR	8–10	No	Physiotherapist
	G2: DN	Yes	Hong	1	Sternocleidomastoid muscle	0.25 × 0.25 mm	1.5cm away the TrP	NR	NR	8–10	No	Physiotherapist
Onat et al. 2019 [26]	G1: DN	Yes	Hong	1	The posterior muscles of the cervical spine	NR	Until TrP	NR	NR	6–8	No	Physician
Tabatabaiee et al. 2019 [47]	G1: DN	Yes	Hong	1	Upper trapezius	0.25 × 40 mm	Until TrP	NR	60 sg	Until a LTR was elicited	Yes	Physiotherapist
Ziaeifar et al. 2019 [24]	G1: DN	Yes	Hong	1	Upper trapezius	0.30 × 50 mm	Until TrP	NR	NR	After eliciting LTR, needling was stopped. If no twitch was elicited, needling was stopped after 2-3 stellate movements	Yes	Therapist
Sukareechai et al. 2019 [48]	G1: DN	Yes	Multiple needle entry technique	NR	Upper trapezius, rhomboid and infraspinatus muscle	0.25 × 50 mm	NR	NR	NR	NR	No	NR
Arias-Buría et al. 2020 [43]	G1: DN	Yes	Hong	1	Anterior scalene muscle	0.30 × 30 mm	Until TrP	25–30 sg	1	Until the first LTR was obtained	Yes	Physiotherapist
García-de-Miguel et al. 2020 [44]	G1: DN	Yes	Hong	1	Levator scapulae	0.25 × 25 mm	Until TrP	NR	NR	8–10 times	No	Physiotherapist
	G2: PENS	Yes	Hong and electrostimulation asymmetric current at a 2-Hz with a pulse width of 100 us	2	Levator scapulae	0.25 × 25 mm	Until TrP	20 min	NR	8–10 times	No	Physiotherapist
Valiente-Castrillo et al. 2020 [45]	G1: DN	Yes	Hong	4	Upper trapezius, levator scapulae, cervical multifidus, and splenius cervicis	032x40 mm	Until TrP	NR	NR	Until to obtain five LTR	Yes	Physiotherapist

DN: dry needling, G: group, and LTR: local twitch response.

**Table 4:** Methodological quality score (Physiotherapy Evidence Database (PEDro) scale) of randomized clinical trials.

Study	1	2	3	4	5	6	7	8	9	10	TOTAL
Ibuldu et al. 2004 [36]	Y	N	Y	Y	N	Y	Y	N	Y	Y	7/10
Itoh et al. 2007 [33]	Y	N	Y	Y	N	Y	N	N	Y	Y	6/10
Myburgh et al. 2012 [27]	Y	N	Y	Y	N	Y	Y	N	Y	Y	7/10
Tekin et al. 2012 [46]	Y	N	Y	Y	N	Y	Y	N	Y	Y	7/10
Llamas-Ramos et al. 2014 [32]	Y	Y	Y	N	N	Y	Y	Y	Y	Y	8/10
Mejuto-Vázquez et al. 2014 [28]	Y	Y	Y	N	N	Y	Y	N	Y	Y	7/10
Ziaeifar et al. 2014 [35]	Y	N	Y	N	N	N	Y	N	Y	Y	5/10
Rayegani et al. 2014 [51]	Y	N	Y	N	N	N	N	N	Y	Y	4/10
Campa-Moran et al. 2015 [41]	Y	N	N	N	N	Y	Y	Y	Y	Y	6/10
Pecos-Martín et al. 2015 [25]	Y	N	Y	Y	N	Y	Y	Y	Y	Y	8/10
Aridici et al. 2016 [42]	Y	Y	Y	N	N	Y	Y	Y	Y	Y	8/10
Hayta et al. 2016 [37]	Y	N	Y	N	N	Y	Y	N	Y	Y	6/10
Segura-Ortí et al. 2016 [50]	Y	Y	Y	N	N	Y	N	N	Y	Y	6/10
Ziaeifar et al. 2016 [23]	Y	N	Y	N	N	N	Y	Y	Y	Y	6/10
Fernández-Carnero et al. 2017 [38]	Y	Y	Y	Y	N	Y	Y	N	Y	Y	8/10
Sobhani et al. 2017 [49]	Y	N	Y	N	N	Y	N	N	Y	Y	5/10
De Meulemeester et al. 2017 [40]	Y	Y	Y	N	N	Y	Y	N	Y	Y	7/10
Dogan et al. 2019 [39]	Y	N	Y	N	N	Y	Y	N	Y	Y	6/10
Luan et al. 2019 [31]	Y	Y	Y	N	N	Y	Y	N	Y	Y	7/10
Manafnezhad et al. 2019 [30]	Y	N	Y	N	N	Y	Y	N	Y	Y	6/10
Martín-Rodríguez et al. 2019 [29]	Y	Y	Y	Y	N	Y	Y	N	Y	Y	8/10
Onat et al. 2019 [26]	Y	Y	Y	N	N	N	Y	Y	Y	Y	7/10
Tabatabaiee et al. 2019 [47]	Y	N	Y	N	N	N	Y	N	Y	Y	5/10
Ziaeifar et al. 2019 [24]	Y	N	Y	N	N	N	Y	N	Y	Y	5/10
Sukareechai et al. 2019 [48]	Y	N	Y	N	N	N	Y	Y	Y	Y	6/10
Arias-Buría et al. 2020 [43]	Y	Y	Y	N	N	Y	Y	Y	Y	Y	8/10
García-de-Miguel et al. 2020 [44]	Y	Y	Y	N	N	Y	Y	Y	Y	Y	8/10
Valiente-Castrillo et al. 2020 [45]	Y	Y	Y	N	N	Y	Y	Y	Y	Y	8/10

(1) Random Allocation of Participants, (2) Concealed Allocation, (3) Similarity Between Groups at Baseline, (4) Participant Blinding, (5) Therapist Blinding, (6) Assessor Blinding, (7) Fewer than 15% Dropouts, (8) Intention-to-Treat Analysis, (9) Between-Group Statistical Comparisons, and (10) Point Measures and Variability Data.

**Table 5:** Adverse events described in the included studies.

Ibuldu et al. 2004 [36]	No data about adverse events were provided.
Itoh et al. 2007 [33]	One patient in the sham group was excluded due to deterioration of symptoms. No adverse events were observed during treatment.
Myburgh et al. 2012 [27]	Within the DN group, 5 patients (29.4%) perceived post-needling soreness, and 8 patients (47.1%) perceived muscle strength soreness (diffuse muscle fatigue) 48 hours postintervention. Within the sham needling group, 9 patients (45%) experienced post-needling soreness.
Tekin et al. 2012 [46]	No data about adverse events were provided.
Rayegani et al. 2014 [51]	No data about adverse events were provided.
Llamas-Ramos et al. 2014 [32]	Twenty-six patients (55%) assigned to DN group experienced post-needling soreness. Eleven patients assigned to manual therapy group experienced muscle fatigue. All minor adverse events resolved spontaneously within 24-48 h without further treatment.
Ziaeifar et al. 2014 [35]	No data about adverse events were provided.
Mejuto-Vázquez et al. 2014 [28]	Eighty-eight percent (88%) of patients assigned in the DN group experienced post-needling soreness. This minor adverse event resolved spontaneously within 24-36 h without further treatment.
Campa-Moran et al. 2015 [41]	No adverse effect was registered after the needling application.
Pecos-Martín et al. 2015 [25]	No data about adverse events were provided.
Aridici et al. 2016 [42]	No data about adverse events were provided.
Segura-Ortí et al. 2016 [50]	Two subjects assigned to the DN group dropped out due to aversion to needles. No other adverse event was observed.
Hayta et al. 2016 [37]	No data about adverse events were provided.
Ziaeifar et al. 2016 [23]	No data about adverse events were provided.
Sobhani et al. 2017 [49]	No data about adverse events were provided.
Fernández-Carnero et al. 2017 [38]	Ninety-one percent (91%) of the patients reported post-needling soreness. No other adverse effects were reported
De Meulemeester et al. 2017 [40]	Post-needling soreness. No other adverse effects were reported.
Luan et al. 2019 [31]	No adverse effects were observed during the study.
Dogan et al. 2019 [39]	No adverse effects were observed during the study.
Manafnezhad et al. 2019 [30]	No data about adverse events were provided.
Martín-Rodríguez et al. 2019 [29]	Within the non-trigger point DN group, three patients (17.6%) experimented contralateral side pain, 4 patients (23.5%) suffered headache, one patient (5.9%) earache, and one (5.9%) hematoma. Within the trigger point DN group, three patients (17.6%) experimented contralateral side pain and one patient (2.9%) post-needling soreness.
Tabatabaiee et al. 2019 [47]	No data about adverse events were provided.
Onat et al. 2019 [26]	Three patients (8.3%) in the DN group experienced an increase in neck pain after dry needling, and 2 patients (5.5%) in the Kinesiotaping group showed cutaneous irritation.
Ziaeifar et al. 2019 [24]	No data about adverse events were provided.
Sukareechai et al. 2019 [48]	Some participants experienced soreness after dry needling therapy.
Arias-Buría et al. 2020 [43]	Six patients assigned to the DN experienced post-needling soreness, but it resolved spontaneously.
Valiente-Castrillo et al. 2020 [45]	Ninety percent (90%) patients presented post-needling soreness after DN, but it resolved spontaneously.
García-de-Miguel et al. 2020 [44]	No data about adverse events were provided.

DN: Dry Needling.

**Table 6:** Level of Evidence (Grading of Recommendations Assessment, Development, and Evaluation (GRADE)) for dry needling on pain intensity, pressure pain sensitivity, and cervical range of motion in patients with neck pain.

Number of Studies	Risk of Bias	Inconsistency	Indirectness of Evidence	Imprecision	Publication Bias	Quality of Evidence	MD or SMD (95% CI)
Dry Needling vs. Sham/Control vs. Physical Therapy Modalities on Neck Pain Intensity							
Immediate Follow-Up (less than 1 week after single session)							
Overall effect (*n* = 11)	No	Serious (I^2^ = 77%)	No	No	No	Moderate	MD −0.75 (−1.43 to −0.06) *
Sham/Placebo/Waiting list/Other form of Dry Needling (*n* = 6)	No	Serious (I^2^ = 58%)	No	Serious	No	Low	MD −1.53 (−2.29 to −0.76) *
Manual Therapy (*n* = 3)	No	No (I^2^ = 0%)	No	Very Serious	No	Low	MD 0.19 (−0.61 to 1.00)
Other Physical Therapy Intervention (*n* = 2)	No	No (I^2^ = 0%)	No	Very Serious	No	Low	MD −0.07 (−0.51 to 0.37)
Short-term Follow-Up (1 to 12 weeks after intervention)							
Overall effect (*n* = 24)	No	Very Serious (I^2^ = 87%)	No	No	No	Low	MD −0.65 (−1.09 to −0.22) *
Sham/Placebo/waiting list/Other form of Dry Needling (*n* = 6)	No	Very Serious (I^2^ = 87%)	No	No	No	Low	MD −2.31 (−3.64 to −0.99) *
Manual Therapy (*n* = 7)	No	Serious (I^2^ = 46%)	No	No	No	Moderate	MD −0.53 (−0.97 to −0.09) *
Other Physical Therapy Intervention (*n* = 13)	No	Serious (I^2^ = 44%)	No	No	No	Moderate	MD 0.10 (−0.21 to 0.41)
Mid-term Follow-Up (more than 12 weeks after intervention)							
Overall effect (*n* = 5)	No	No (I^2^ = 28%)	No	Very Serious	No	Low	MD −0.27 (−0.73 to 0.18)
Manual Therapy (v = 2)	No	No (I^2^ = 0%)	No	Very Serious	No	Low	MD −0.75 (−1.70 to 0.20)
Other Physical Therapy Intervention (*n* = 3)	No	Serious (I^2^ = 47%)	No	Very Serious	No	Very Low	MD −0.19 (−0.75 to 0.38)
Dry Needling vs. Sham/Control vs. Physical Therapy Modalities on Pain-Related Disability							
Short-term Follow-Up (1 to 12 weeks after intervention)							
Overall effect (*n* = 20)	No	Serious (I^2^ = 58%)	No	No	Yes	Low	SMD −0.26 (−0.48 to −0.05) *
Sham/Placebo/Waiting list/Other form of Dry Needling (*n* = 5)	No	Serious (I^2^ = 79%)	No	No	No	Moderate	SMD −0.87 (−1.60 to −0.14) *
Manual Therapy (*n* = 7)	No	No (I^2^ = 23%)	No	No	No	High	SMD −0.20 (−0.49 to 0.10)
Other Physical Therapy Intervention (*n* = 9)	No	No (I^2^ = 12%)	No	No	No	High	SMD −0.07 (−0.27 to 0.13)
Mid-term Follow-Up (more than 12 weeks after intervention)							
Overall effect (*n* = 5)	No	Serious (I^2^ = 48%)	No	Very Serious	No	Very Low	SMD −0.33 (−0.70 to 0.05)
Manual Therapy (*n* = 2)	No	No (I^2^ = 0%)	No	Very Serious	No	Low	SMD −0.40 (−0.88 to 0.08)
Other Physical Therapy Intervention (*n* = 3)	No	Serious (I^2^ = 71%)	No	Very Serious	No	Very Low	SMD −0.29 (−0.88 to 0.31)
Dry Needling vs. Sham/Control vs. Physical Therapy Modalities on Pressure Pain Thresholds							
Immediate Follow-Up (less than 1 week after single session)							
Overall effect (*n* = 9)	No	Very Serious (I^2^ = 92%)	No	No	No	Low	MD 4.93 (−42.18 to 52.04)
Sham/Placebo/Waiting list/Other form of dry needling (*n* = 4)	No	No (I^2^ = 28%)	No	Serious	No	Moderate	MD 55.48 (27.03 to 83.93) *
Manual Therapy (*n* = 3)	No	No (I^2^ = 0%)	No	Very Serious	No	Low	MD −11.77 (−37.02 to 13.47)
Other Physical Therapy Intervention (*n* = 2)	No	Very Serious (I^2^ = 97%)	No	Very Serious	No	Very Low	MD −58.34 (−159.05 to 42.38)
Short-term Follow-Up (1 to 12 weeks after intervention)							
Overall effect (*n* = 17)	No	Very Serious (I^2^ = 95%)	No	No	No	Low	MD 6.84 (−33.41 to 47.12)
Sham/Placebo/Waiting list/Other form of dry needling (*n* = 3)	No	Very Serious (I^2^ = 90%)	No	Very Serious	No	Very Low	MD 75.53 (−9.82 to 160.88)
Manual Therapy (*n* = 6)	No	Serious (I^2^ = 75%)	No	No	No	Moderate	MD 32.25 (−1.02 to 65.52)
Other Physical Therapy Intervention (*n* = 8)	No	Very Serious (I^2^ = 93%)	No	No	No	Low	MD −33.89 (−89.44 to 21.66)
Number of studies	Risk of bias	Inconsistency	Indirectness of evidence	Imprecision	Publication bias	Quality of evidence	MD or SMD (95% CI)
Dry Needling vs. Sham/Control vs. Physical Therapy Modalities on Cervical Range of Motion							
Cervical Flexion (Immediate Follow-Up, less than 1 week after single session)							
Overall effect (*n* = 5)	No	Very Serious (I^2^ = 83%)	No	Very Serious	No	Very Low	MD 1.93 (−5.90, 9.77)
Sham/Placebo/Waiting list/Other form of dry needling (*n* = 3)	No	Very Serious (I^2^ = 83%)	No	Very Serious	No	Very Low	MD 7.51 (−3.23, 18.25)
Manual Therapy (*n* = 1)	No	No	No	Very Serious	No	Low	MD −6.43 (−17.96, 5.10)
Other Physical Therapy Intervention (*n* = 1)	No	No	No	Very Serious	No	Low	MD −6.37 (−11.09, −1.65)
Cervical Flexion (Short-term Follow-Up, 1 to 12 weeks after intervention)							
Overall effect (*n* = 10)	No	Very Serious (I^2^ = 85%)	No	No	No	Low	MD 1.26 (−3.06, 5.58)
Sham/Placebo/Waiting list/Other form of dry needling (*n* = 3)	No	Very Serious (I^2^ = 87%)	No	Very Serious	No	Very Low	MD 6.76 (−4.53, 18.06)
Manual Therapy (*n* = 3)	No	No (I^2^ = 0%)	No	Very Serious	No	Low	MD 0.26 (−2.07, 2.60)
Other Physical Therapy Intervention (*n* = 4)	No	Very Serious (I^2^ = 92%)	No	Very Serious	No	Very Low	MD −1.74 (−10.51, 7.03)
Cervical Extension (Immediate Follow-Up, less than 1 week after single session)							
Overall effect (*n* = 5)	No	Serious (I^2^ = 63%)	No	Very Serious	No	Very Low	MD 5.23 (−1.05, 11.51)
Sham/Placebo/Waiting list/Other form of dry needling (*n* = 3)	No	Serious (I^2^ = 77%)	No	Very Serious	No	Very Low	MD 8.70 (−1.94, 19.35)
Manual Therapy (*n* = 1)	No	No	No	Very Serious	No	Low	MD 1.95 (−11.47, 15.37)
Other Physical Therapy Intervention (*n* = 1)	No	No	No	Very Serious	No	Low	MD 0.68 (−5.01, 6.37)
Cervical Extension (Short-term Follow-Up, 1 to 12 weeks after intervention)							
Overall effect (*n* = 10)	No	Serious (I^2^ = 72%)	No	No	Yes	Low	MD 0.34 (−3.02, 3.70)
Sham/Placebo/Waiting list/Other form of dry needling (*n* = 3)	No	Serious (I^2^ = 66%)	No	Very Serious	No	Very Low	MD 8.26 (−1.36, 17.88)
Manual Therapy (*n* = 3)	No	No (I^2^ = 0%)	No	Very Serious	No	Low	MD −0.21 (−2.70, 2.28)
Other Physical Therapy Intervention (*n* = 4)	No	Very Serious (I^2^ = 85%)	No	Very Serious	No	Very Low	MD −3.04 (−9.94, 3.87)
Cervical Lateral-Flexion (Immediate Follow-Up, less than 1 week after single session)							
Overall effect (*n* = 4)	No	Serious (I^2^ = 67%)	No	Very Serious	No	Very Low	MD 2.65 (−2.07, 7.37)
Sham/Placebo/Waiting list/Other form of dry needling (*n* = 3)	No	Serious (I^2^ = 57%)	No	Very Serious	No	Very Low	MD 4.54 (−1.41, 10.48)
Other Physical Therapy Intervention (*n* = 1)	No	No	No	Very Serious	No	Low	MD −0.95 (−2.07, 7.37)
Cervical Lateral-Flexion (Short-term Follow-Up, 1 to 12 weeks after intervention)							
Overall effect (*n* = 10)	No	Very Serious (I^2^ = 86%)	No	No	No	Low	MD 0.30 (−1.00, 1.61)
Sham/Placebo/Waiting list/Other form of dry needling (*n* = 3)	No	Serious (I^2^ = 66%)	No	Very Serious	No	Very Low	MD 3.21 (−2.28, 8.70)
Manual Therapy (*n* = 3)	No	Serious (I^2^ = 77%)	No	Very Serious	No	Very Low	MD 1.09 (−1.11, 3.28)
Other Physical Therapy Intervention (*n* = 6)	No	No (I^2^ = 25%)	No	No	No	High	MD −0.58 (−1.59, 0.42)
Cervical Rotation (Immediate Follow-Up, less than 1 week after single session)							
Overall effect (*n* = 4)	No	Serious (I^2^ = 60%)	No	Very Serious	No	Very Low	MD 2.04 (−4.08, 8.15)
Sham/Placebo/Waiting list/Other form of dry needling (*n* = 3)	No	No (I^2^ = 22%)	No	Very Serious	No	Low	MD 4.51 (−0.96, 9.98)
Other Physical Therapy Intervention (*n* = 1)	No	No	No	Very Serious	No	Low	MD −3.77 (−9.58, 2.04)
Cervical Rotation (Short-term Follow-Up, 1 to 12 weeks after intervention)							
Overall effect (*n* = 9)	No	Serious (I^2^ = 74%)	No	No	Yes	Low	MD −0.23 (−1.40, 1.09)
Sham/Placebo/Waiting list/Other form of dry needling (*n* = 3)	No	Serious (I^2^ = 71%)	No	Very Serious	No	Very Low	MD 6.20 (−1.08, 13.48)
Manual Therapy (*n* = 3)	No	Serious (I^2^ = 70%)	No	Very Serious	No	Very Low	MD −0.52 (−1.91, 0.87)
Other Physical Therapy Intervention (*n* = 5)	No	Serious (I^2^ = 47%)	No	Very Serious	No	Very Low	MD −0.82 (−2.73, 1.09)

* Statistically significant (*p* < 0.05). Risk of bias: No: Most information is from results at a low risk of bias. Serious: Crucial limitation for one criterion, or some limitations for multiple criteria, sufficient to lower confidence in the estimate of the effect. and Very Serious: Crucial limitation for one or more criteria sufficient to substantially lower the confidence in the estimate of the effect. Inconsistency: Serious: I^2^ > 40% and Very Serious: I^2^ > 80%. Indirectness of Evidence: No indirectness of evidence was found in any study. Imprecision (based on the sample size): Serious: *n* < 250 subjects and Very Serious: *n* < 250 and the estimated effect is little or absent. Publication bias (based on funnel plots): Funnel plots are shown as Supplementary Files in those analyses with more than 10 trials. MD: mean differences and SMD: standardized mean differences.

## Supplementary Materials

The following are available online at https://www.mdpi.com/2077-0383/9/10/3300/s1: Figure S1: Funnel Plot of the trials (*n* = 11) investigating the immediate effects of dry needling on pain intensity. The funnel plot showed small asymmetry not associated to potential publication bias. Figure S2: Funnel Plot of the trials (*n* = 11) investigating the immediate effects of dry needling on pain intensity. The funnel plot showed small asymmetry not associated to potential publication bias. Figure S3: Funnel Plot of the trials (*n* = 20) investigating the short-term effects of dry needling on pain-related disability. The funnel plot showed asymmetry due to the study by Itoh et al 2007 [33], therefore, it was associated to potential publication bias. The exclusion of this study would tend to a symmetric funnel plot. Figure S4: Funnel Plot of the trials (*n* = 17) investigating the short-term effects of dry needling on pressure pain thresholds. The funnel plot showed small asymmetry not associated to potential publication bias. Table S1: Main results and raw data of the included studies
