# Supplementary material for: Effectiveness of Dry Needling for Myofascial Trigger Points Associated with Neck Pain Symptoms: An Updated Systematic Review and Meta-Analysis
Source: J Clin Med. 2020 Oct 14;9(10):3300. doi: 10.3390/jcm9103300 (PMC7602246; doi:10.3390/jcm9103300)
Supplement: Supplementary file 1 [file jcm-09-03300-s001.pdf]

Journal of
Clinical Medicine

MDPI

Supplementary Material

Table 1. Main results and raw data of the included studies.

Study	Outcome/Group	Baseline Mean (SD)	Immediate, Less 1 Week After a Single Session Mean (SD)	Short-Term, 1 to 12 Weeks After Treatment Period Mean (SD)	Mid-Term, 12 to 24 Weeks Mean (SD)
	Pain (VAS, 0-10)				
	G1	5.10 (1.97)		3.71 (2.33) (1wk)	
	G2	5.50 (1.96)		2.05 (1.43) (1wk)	
	G3 (N/A)	5.70 (1.81)		3.65 (2.03) (1wk)	
	PPT (kPa)				
Ilbuldu et al. 2004	G1	214.76 (93.16)		246.14 (153.96) (1wk)	
	G2	264.77 (69.62)		391.28 (119.64) (1wk)	
	G3 (N/A)	248.10 (40.20)		240.26 (60.80) (1wk)	
	Cervical Flexion (º)				
	G1	51.24 (7.60)		59.67 (10.52) (1wk)	
	G2	56.10 (10.3)		64.15 (9.25) (1wk)	
	G3 (N/A)	55.60 (10.4)		50.84 (10.25) (1wk)	
	Cervical Extension (º)				
	G1	60.90 (15.26)		72.86 (12.18) (1wk)	
	G2	65.05 (9.39)		81.95 (10.84) (1wk)	
	G3 (N/A)	63.90 (14.48)		65.70 (13.93) (1wk)	
	Right Cervical Lateral Flexion (º)				
	G1	34.19 (7.63)		41.71 (10.05) (1wk)	
	G2	34.40 (12.18)		52.65 (10.23) (1wk)	
	G3 (N/A)	37.20 (7.05)		41.45 (10.00) (1wk)	
	Left Cervical Lateral Flexion (º)				
	G1	38.10 (8.46)		49.00 (11.29) (1wk)	
	G2	32.80 (10.88)		53.90 (12.35) (1wk)	
Ilbuldu et al. 2004	G3 (N/A)	37.45 (9.42)		44.20 (9.66) (1wk)	
	Cervical Lateral Flexion (mean calculated)				
	G1	36.14 (8.04)		45.35 (10.67) (1wk)	
	G2	33.6 (11.53)		53.27 (11.29) (1wk)	

	G3 (N/A)	37.32 (8.23)		44.82 (9.83) (1wk)
	Right Cervical Rotation (º)			
	G1	63.71 (16.27)		78.52 (9.94) (1wk)
	G2	60.00 (14.60)		79.25 (9.98) (1wk)
	G3 (N/A)	62.90 (8.84)		73.05 (11.46) (1wk)
	Left Cervical Rotation (º)			
	G1	69.00 (12.58)		77.90 (9.94) (1wk)
	G2	62.45 (15.80)		80.60 (13.19) (1wk)
	G3 (N/A)	66.85 (10.94)		73.80 (10.74) (1wk)
	Cervical Rotation (calculated mean)			
	G1	63.35 (14.42)		78.21 (9.94) (1wk)
	G2	61.22 (15.2)		79.92 (11.58) (1wk)
	G3 (N/A)	64.87 (9.89)		73.42 (11.1) (1wk)
Itoh et al. 2007	Pain (VAS, 0-10)			
	G1	6.7 (1.32)		1.1 (0.93) (2wk)
	G2	7.09 (1.4)		5.76 (1.8) (2wk)
	G3 (N/A)	6.41 (2.07)		5.39 (2.3) (2wk)
	G4 (N/A)	6.95 (1.86)		5.16 (2.2) (2wk)
	Disability (NDI)			
	G1	13.0 (6.3)		3.1 (3.2) (2wk)
	G2	15.1 (2.7)		12.0 (4.5) (2wk)
	G3 (N/A)	12.0 (3.6)		11.1 (5.09) (2wk)
	G4 (N/A)	12.6 (6.0)		10.9 (6.6) (2wk)
Myburgh et al. 2012	Pain (NPRS, 0-10)			
	G1	5.59 (4.69-6.50)	3.41 (2.31-4.52) (48h)	
	G2	5.40 (4.48 -6.32)	4.60 (3.62-5.58) (48h)	
	PPT (kPa)			
	G1	216.58 (120.27)	186.48 (124.19) (48h)	
	G2	168.76 (72.60)	138.55 (28.42) (48h)	
Tekin et al. 2012	Pain (VAS, 0-10)			
	G1	6.6 (1.3)	4.0 (1.6) (48h)	2.2 (2.0) (4wk)
	G2	6.4 (1.6)	5.4 (1.6) (48h)	5.3 (1.8) (4wk)
Rayegani et al. 2014	Pain (VAS, 0-10)			
	G1	2.9 (2.8)		1.5 (2.8) (4wk)
	G2	3.6 (2.6)		2.2 (2.6) (4wk)

	PPT (kPa)	266 (72)	374 (101) (4wk)
	G1	255 (82)	328 (94) (4wk)
	G2		
	Pain (NPRS, 0-10)		
	G1	6.2 (1)	0.9 (0.8) (2wk)
	G2	6.2 (1.3)	1.0 (1.1) (2wk)
	Disability (NPQ)		
	G1	19.1 (6.4)	5.4 (3.1) (2wk)
	G2	17.8 (7.3)	5.0 (3.7) (2wk)
	PPT (kPa)		
	G1	188.1 (39.5)	326.2 (39.0) (2wk)
	G2	188.1 (49.4)	247.3 (49.0) (2wk)
	Cervical Flexion (º)		
	G1	54.1 (7.0)	60.2 (6.6) (2wk)
	G2	54.2 (6.9)	59.8 (6.0) (2wk)
Llamas-Ramos et al. 2014			
	Cervical Extension (º)		
	G1	60.5 (8.02)	68.0 (8.02) (2wk)
	G2	60.7 (7.5)	68.2 (5.1) (2wk)
	Cervical Lateral Flexion (toward treated side) (º)		
	G1	38.8 (3.6)	43.2 (3.8) (2wk)
	G2	39.9 (5.0)	42.9 (3.7) (2wk)
	Cervical Lateral Flexion (away from treated side) (º)		
	G1	38.6 (3.9)	45.4 (3.6) (2wk)
	G2	38.5 (4.95)	44.9 (4.7) (2wk)
	Cervical Lateral Flexion (mean calculated)		
	G1	38.7 (3.75)	44.3 (3.7) (2wk)
	G2	39.2 (4.95)	44.9 (4.7) (2wk)
	Cervical Rotation (º) (toward treated side)		
Llamas-Ramos et al. 2014	G1	63.5 (5.9)	70.0 (2.4) (2wk)
	G2	64.4 (5.5)	70.0 (3.0) (2wk)

	Cervical Rotation (º) (away from treated side)			
	G1	65.8 (5.4)		70.5 (2.4) (2wk)
	G2	66.6 (5.8)		69.6 (2.9) (2wk)
	Cervical Rotation (mean calculated)			
	G1	64.65 (5.65)		70.25 (2.4) (2wk)
	G2	65.5 (5.65)		69.8 (2.95) (2wk)
Ziaeifar et al. 2014	Pain (VAS, 0-10)			
	G1	6.56 (1.53)		1.34 (1.93) (1wk)
	G2	6.23 (1.26)		3.05 (2.27) (1wk)
	PPT (kPa)			
	G1	104.041 (39.324)		161.12 (45.69) (1wk)
	G2	106.597 (38.441)		142.19 (43.54) (1wk)
Mejuto-Vázquez et al. 2014	Pain (NPRS, 0-10)			
	G1	5.7 (1.8)	3.8 (1.9) (10min)	2.0 (1.7) (1wk)
	G2	5.3 (2.0)	5.5 (2.1) (10 min)	4.6 (2.1) (1wk)
	PPT (kPa)			
	G1	224.8 (100.3)	294.0 (69.6) (10 min)	353.7 (92.3) (1wk)
	G2	285.1 (102.4)	282.0 (109.5) (10 min)	296.1 (89.7) (1wk)
	Cervical Flexion (º)			
	G1	58.3 (12.2)	68.3 (13.4) (10min)	67.7 (5.0) (1wk)
	G2	51.2 (9.9)	46.2 (9.5) (10min)	50.6 (7.7) (1wk)
	Cervical Extension (º)			
	G1	65.0 (16.2)	78.8 (12.6) (10min)	80.5 (8.4) (1wk)
	G2	61.8 (13.6)	58.1 (16.0) (10min)	60.2 (16.3) (1wk)
	Cervical Lateral Flexion (toward treated side) (º)			
	G1	36.1 (13.4)	48.3 (6.6) (10min)	51.6 (4.3) (1wk)
	G2	38.7 (12.7)	38.1 (10.0) (10min)	41.2 (10.3) (1wk)
	Cervical Lateral Flexion (away from treated side) (º)			
	G1	39.4 (15.1)	51.6 (9.3) (10min)	52.2(6.6) (1wk)
	G2	36.2 (11.5)	37.5 (12.8) (10min)	42.5 (8.5) (1wk)
	Cervical Lateral Flexion (mean calculated)			
	G1	37.75 (14.25)	49.95 (7.95) (10min)	51.9 (5.45) (1wk)
	G2	37.45 (12.1O	37.8 (11.4) (10min)	41.85 (9.4) (1wk)
	Cervical Rotation (º) (toward treated side)			

Mejuto-Vázquez et al. 2014	G1	55.5 (24.1)	66.6 (15.6) (10min)	72.7 (14.1) (1wk)
	G2	58.1 (15.1)	53.7 (15.0) (10min)	57.5 (7.0) (1wk)
	Cervical Rotation (º) (away from treated side)			
	G1	58.8 (23.5)	68.3 (15.4) (10min)	74.4 (8.0) (1wk)
	G2	59.3 (18.2)	55.1 (18.2) (10min)	58.7 (7.9) (1wk)
	Cervical Rotation (mean calculated)			
	G1	57.15 (23.8)	67.45 (15.5) (10min)	73.55 (11.05) (1wk)
	G2	58.7 (16.65)	54.4 (16.6) (10min)	58.1 (7.45) (1wk)
Campa-Moran et al. 2015	VAS Pain (0-10)†			
	G1	3.38 (1.17)	3.97 (1.77) (10min)	1.33 (1.47) (1wk)
	G2	5.02 (1.77)	3.82 (1.92) (10min)	3.43 (1.47) (1wk)
	G3	4.21 (1.63)	2.98 (1.77) (10min)	0.94 (1.43) (1wk)
	G2 + G3 (n=24)	4.61 (1.71)	3.4 (1.85)	2.18 (1.90)
	Disability (NDI)			
	G1	18.0 (5.4)		12.2 (5.66) (1wk)
	G2	17.4 (4.8)		15.2 (5.66) (1wk)
	G3	18.5 (3.2)		10.0 (5.50) (1wk)
	G2 + G3 (*n* = 24)	17.95 (4.02)		12.6 (6.06)
	PPT (kPa)†			
	G1	292.93 (294.18)	295.48 (188.32) (10min)	316.47 (306.75) (1wk)
	G2	228.04 (107.72)	172.17 (129.11) (10min)	249.92 (152.96) (1wk)
	G3	257.18 (133.22)	344.66 (109.37) (10min)	398.23 (51.80) (1wk)
	G2 + G3 (*n* = 24)	242.61 (119.41)	258.41 (146.47)	324.07 (134.94)
	Cervical Flexion (º)†			
	G1	38.04 (3.68)	39.48 (16.07) (10min)	44.78 (7.88) (1wk)
	G2	40 (15.76)	46.45 (19.37) (10min)	41.04 (14.67) (1wk)
	G3	39.63 (13.41)	45.18 (16.77) (10min)	53.41 (10.48) (1wk)
	G2 + G3 (*n* = 24)	39.81 (14.31)	45.91 (17.73)	47.22 (13.97)
	Cervical Extension (º) †			
	G1	43.39 (14.50)	49.96 (16.26) (10min)	50.62 (15.34) (1wk)
	G2	43.41 (27.25)	40.54 (16.26) (10min)	48.64 (4.27) (1wk)
	G3	53.32 (25.15)	55.49 (29.35) (10min)	60.43 (5.19) (1wk)
	G2 + G3 (*n* = 24)	48.36 (26.12)	48.01 (24.42)	54.53 (7.60)
	Pain (NPRS, 0-10)			
	G1			

Pecos-Martin et al 2015	G2	5.3 (1.5)	2.6 (1.8) (1wk)	2.1 (1.6) (4wk)	
		5.6 (1.6)	5.3 (1.6) (1wk)	5.1 (1.5) (4wk)	
	Disability (NPQ)				
	G1				
	G2	22.1 (11.4)		9.9 (7.4) (4wk)	
		19.7 (7.9)		19.7 (7.9) (4wk)	
	PPT (kPa)				
	G1				
	G2	274.58 (49.03)	421.68 (58.83) (1wk)	431.49 (49.03) (4wk)	
		274.58 (58.83)	343.23 (68.64) (1wk)	294.19 (58.83) (4wk)	
Aridici et al. 2016	Pain (VAS, 0-10)				
	G1	7.80 (1.72)		4.58 (1.85) (1wk)	
	G2	7.66 (1.66)		4.20 (2.05) (1wk)	
	Disability (NPDS)				
	G1	66.54 (16.84)		42.74 (20.95) (1wk)	
	G2	66.0 (14.46)		39.56 (19.77) (1wk)	
Segura-Ortí et al. 2016	Pain (VAS, 0-100)				
	G1	36.2 (22.5)		17.7 (14.7) (1wk)	
	G2	46.9 (20.9)		18.6 (10.3) (1wk)	
	G3 (N/A)	34.2 (17.5)		12.3 (9.3) (1wk)	
	PPT (kPa)				
	G1	215.8 (78.5)		245.2 (78.5) (1wk)	
	G2	215.8 (29.4)		245.2 (29.4) (1wk)	
	G3 (N/A)	196.1 (29.4)		225.6 (58.8) (1wk)	
	Disability (NDI)				
	G1	7.2 (3.4)		5.8 (4.2) (1wk)	
	G2	10.2 (7.7)		4.8 (3.1) (1wk)	
	G3 (N/A)	8.8 (4.0)		7.0 (3.7) (1wk)	
Hayta et al. 2016	Pain (VAS,0-10)				
	G1	7.1 (1.2)		5.5 (1.2) (4wk)	3.8 (1.1) (12wk)
	G2	7.1 (1.2)		5.7 (1.2) (4wk)	4.2 (1.3) (12wk)
	Disability (NDI)				
	G1	18.4 (7.0)		12.6 (5.6) (4wk)	7.6 (3.7) (12wk)
	G2	19.7 (5.8)		15.9 (5.6) (4wk)	11.1 (5.3) (12wk)
Ziaeifar et al. 2016	Pain (NPRS,0-10)				
	G1	7.96 (1.52)	7.85 (2.24) † (48h)		

	G2	8.26 (1.71)	7.55 (2.17) † (48h)	
	PPT (kPa)			
	G1	104.05 (39.32)	102.57 (47.85) † (48h)	
	G2	106.59 (38.24)	123.17 (42.75) † (48h)	
	Pain (VAS, 0-10)			
	G1	5.00 (1.89)	3.76 (1.70) (72h)	3.05 (1.98) (1wk)
	G2	4.76 (1.72)	2.43 (1.69) (72h)	2.67 (2.30) (1wk)
Fernández-Carnero et al.	G3	4.81 (1.82)	2.48 (1.88) (72h)	2.24 (2.30) (1wk)
2017	G1 + G2 + G3 (*n*=63)	4.85 (1.77)	2.88 (1.83) (72h)	2.65 (2.09) (1wk)
	G4	4.81 (1.86)	3.33 (2.45) (72h)	3.52 (2.35) (1wk)
	Disability (NDI)			
	G1	12.76 (7.09)		7.05 (3.51) (1wk)
	G2	12.14 (4.94)		7.14 (4.44) (1wk)
	G3	11.05 (5.01)		7.19 (5.95) (1wk)
	G1 + G2 + G3 (*n*=63)	11.98 (5.71)		7.12 (4.66) (1wk)
	G4	11.62 (5.94)		8.19 (6.00) (1wk)
	PPT (kPa)			
	G1	176.51 (93.16)	174.55 (110.81) (72h)	183.38 (85.31) (1wk)
	G2	176.51 (59.82)	182.40 (73.54) (72h)	187.30 (108.85) (1wk)
	G3	204.95 (100.02)	236.33 (152.98) (72h)	243.37 (137.29) (1wk)
	G1 + G2 + G3 (*n*=63)	185.99 (85.80)	197.75 (118.41) (72h)	204.68 (114.07) (1wk)
	G4	178.48 (90.22)	163.77 (85.31) (72h)	181.42 (85.31) (1wk)
Fernández-Carnero et al.				
2017	Cervical Flexion (º)			
	G1	48.66 (11.62)	49.06 (10.44) (72h)	47.61 (11.49) (1wk)
	G2	51.44 (7.85)	54.92 (7.82) (72h)	52.65 (8.09) (1wk)
	G3	48.41 (10.66)	49.76 (11.02) (72h)	50.79 (11.03) (1wk)
	G1 + G2 + G3 (*n* = 63)	49.50 (10.09)	51.24 (10.04) (72h)	50.35 (10.36) (1wk)
	G4	47.87 (12.11)	47.16 (9.09) (72h)	49.20 (10.81) (1wk)
	Cervical Extension (º)			
	G1	58.15 (18.83)	58.03 (13.93) (72h)	62.85 (14.79) (1wk)
	G2	59.25 (13.96)	62.82 (13.07) (72h)	64.12 (12.71) (1wk)
	G3	58.47 (13.18)	58.73 (14.69) (72h)	61.03 (13.55) (1wk)
	G1 + G2 + G3 (*n* = 63)	58.62 (15.27)	59.85 (13.84) (72h)	62.66 (13.54) (1wk)
	G4	59.52 (16.60)	60.22 (13.97) (72h)	60.06 (14.97) (1wk)
	Lateral Flexion (º)			
	G1	36.11 (8.05)	37.80 (5.35) (72h)	38.57 (6.7) (1wk)
	G2	39.27 (5.16)	53.27 (7.72) (72h)	41.70 (5.58) (1wk)

Fernández-Carnero et al. 2017	G3	39.76 (7.95)	40.76 (6.89) (72h)	41.13 (7.55) (1wk)	
	G1 + G2 + G3 (n = 63)	38.38 (8.01)	43.94 (9.45) (72h)	40.46 (6.69) (1wk)	
	G4	38.79 (9.67)	39.58 (9.45) (72h)	40.17 (9.48) (1wk)	
	Cervical Rotation (º)				
	G1	58.44 (11.8)	59.76 (8.69) (72h)	58.83 (9.85) (1wk)	
	G2	62.78 (6.60)	66.36 (6.68) (72h)	66.51 (7.75) (1wk)	
	G3	60.10 (8.83)	62.09 (7.52) (72h)	62.96 (6.71) (1wk)	
	G1 + G2 + G3 (n = 63)	60.44 (9.34)	62.73 (8.03) (72h)	62.76 (8.66) (1wk)	
	G4	59.76 (9.60)	57.25 (12.08) (72h)	58.58 (11.61) (1wk)	
De Meulemeester et al. 2017	Pain (NPRS, 0-10)				
	G1	4.70 (1.81)			3.59 (2.06) (12wk)
	G2	5.86 (1.36)			4.19 (1.97) (12wk)
	Disability (NDI)				
	G1	11.00 (5.12)		7.71 (4.66) (4wk)	8.06 (5.08) (12wk)
	G2	13.14 (4.60)		10.95 (4.63) (4wk)	9.09 (4.35) (12wk)
	PPT (kPa)				
	G1	196.2 (72.6)	164.6 (69.7) (10 min)	240.01 (84.5) (4wk)	
	G2	162 (59.6)	165.9 (68.7) (10 min)	214.7 (81.8) (4wk)	
Sobhani et al. 2017	Pain (VAS, 0-100)				
	G1	56.1 (19.3)		39.2 (20) (5wk)	
	G2	53.8 (16.0)		33.8 (12.6) (5wk)	
	G3	61.5(18.1)		36.9 (14.9) (5wk)	
	Disability (NDI)				
	G1	21.6 (4.8)		16.7 (3.9) (5wk)	
	G2	24.4 (7.6)		19.6 (6.5) (5wk)	
	G3	26.6 (7.8)		21.4 (6.0) (5wk)	
	Cervical Flexion (º)				
	G1	49.2 (8.8)		55.1 (7.6) (5wk)	
	G2	47.7 (11.6)		52.7 (10.8) (5wk)	
	G3	46.7 (9.3)		50.6(10.2) (5wk)	
	Cervical Extension (º)				
Sobhani et al. 2017	G1	49.4 (8.0)		53.1 (7.6) (5wk)	
	G2	46.8 (8.7)		51.1 (8.4) (5wk)	
	G3	47.6 (10.3)		53.5 (8.8) (5wk)	
	Right Cervical Lateral Flexion (º)				
	G1	37.6 (6.0)		41.9 (6.3) (5wk)	
	G2	35.4 (6.8)		39.5 (6.5) (5wk)	

	G3	35.3 (6.3)		39.9 (6.0) (5wk)	
	Left Cervical Lateral Flexion (º)				
	G1	37.1 (5.5)		39.8 (5.5) (5wk)	
	G2	34.1 (6.4)		37.2 (6.1) (5wk)	
	G3	32.5 (6.1)		35.6 (5.8) (5wk)	
	Cervical Lateral Flexion (mean calculated)				
	G1	37.35 (5.75)		40.85 (5.9) (5wk)	
	G2	34.75 (6.6)		38.35 (6.3) (5wk)	
	G3	33.9 (6.2)		37.75 (5.9) (5wk)	
	Right Cervical Rotation (º)				
Sobhani et al. 2017	G1	75.3 (7.5)		77.8 (7.3) (5wk)	
	G2	75.0 (9.9)		83.3 (8.6) (5wk)	
	G3	73.1 (5.3)		74.4(4.9) (5wk)	
	Left Cervical Rotation (º)				
	G1	75.0 (6.5)		77.5 (6.1) (5wk)	
	G2	74.7 (8.7)		82.3 (6.8) (5wk)	
	G3	72.2 (4.8)		74.5 (5.2) (5wk)	
	Cervical Rotation (º) (mean calculated)				
	G1	75.15 (7)		77.65 (6.7) (5wk)	
	G2	74.85 (9.35)		82.8 (7.7) (5wk)	
	G3	72.65 (5.05)		74.45 (5.05) (5wk)	
	Pain (VAS, 0-10)				
	G1	3.78 (1.18)	2.78 (1.07) (10min)	1.91 (1.00) (1mo)	1.69 (1.03) (12wk)
	G2	3.57 (1.04)	2.93 (0.94) (10min)	1.73 (0.91) (1mo)	1.50 (0.82) (12wk)
	PPT (kPa)		)		
	G1	198.63 (35.16)	246.03 (46.91) (10min)	317.09 (49.82) (1mo)	320.84 (46.08) (12wk)
	G2	206.90 (34.24)	253.10 (51.26) (10min	320.13 (61.15) (1mo)	316.97 (52.37) (12wk)
Luan et al. 2019					
	Disability (NDI)				
	G1	15.31 (2.44)		9.38 (2.46) (1mo)	9.47 (1.87) (12wk)
	G2	15.37 (2.04)		9.57 (1.77) (1mo)	9.07 (1.70) (12wk)
	Pain (VAS, 0-100)				
	G1	36.7 (25.6)		10.75 (3.39, 18.11) (4wk)	
	G2	45.4 (28.1)		16.98 (7.92, 26.60) (4wk)	
	PPT (kPa)				

	G1	313.81 (78.45)		382.4 (323.6, 441.2) (4wk)
	G2	294.19 (107.87)		402 (343.2, 451.1) (4wk)
Dogan et al. 2019				
	Cervical Lateral Flexion (º)			
	G1	40 (30-50)		45.8 (43.2, 48.8) (4wk)
	G2	40 (25-50)		46.7 (44.1, 49.4) (4wk)
	Disability (NDI, %)			
	G1	32.6 (13.8)		15.3 (10.2, 20.4) (4wk)
	G2	31.2 (11.3)		14.8 (10.25, 18.8) (4wk)
	Pain (VAS, 0-10)			
	G1	7.5 (1.7)		3.79 (2.20) (1wk)
	G2	7.3 (1.3)		3.89 (2.16) (1wk)
	Disability (NDI)	28.2 (10.2)		15.29 (8.85) (1wk)
	G1	25.6 (11.3)		16.75 (11.69) (1wk)
	G2			
Manafnezhad et al. 2019				
	PPT (kPa)			
	G1	225.55 (72.56)		338.32 (100.02) [a] (1wk)
	G2	231.43 (94.14)		314.79 (113.75) [a] (1wk)
	Pain (VAS, 0-10)			
	G1	2.5 (1.86)	2.5 (2.16) (24h)	1.7 (2.16) (4wk)
	G2	3.6 (2.20)	3.9 (2.45) (24h)	1.4 (1.53) (4wk)
	Disability (NDI)			
	G1	17.1 (12.23)		11.9 (9.02) (4wk)
	G2	23.2 (13.83)		16.0 (12.85) (4wk)
Martín-Rodríguez et al, 2019				
	Cervical Flexion (º)			
	G1	55.0 (9.68)	51.9 (11.41) (24h)	54.2 (14.57) (4wk)
	G2	55.1 (11.26)	49.4 (9.97) (24h)	52.8 (14.57) (4wk)
	Cervical Extension (º)			
	G1	63.4 (14.10)	66.8 (12.11) (24h)	63.8 (13.47) (4wk)
	G2	59.7 (14.179	57.1 (9.35) (24h)	58.7 (14.84) (4wk)
	Right Cervical Lateral Flexion (º)			
	G1	36.8 (6.00)	38.4 (10.89) (24h)	38.6 (7.40) (4wk)
	G2	41.0 (11.60)	38.6 (9.42) (24h)	38.5 (7.66) (4wk)
	Left Cervical Lateral Flexion (º)			
	G1	40.4 (6.53)	41.1 (10.93) (24h)	41.8 (7.93) (4wk)

	G2	45.7 (9.32)	42.2 (7.28) (24h)	40.3 (8.09) (4wk)
	Cervical Lateral Flexion (mean calculated)			
	G1	38.6 (6.26)	39.75 (10.91) (24h)	40.2 (7.66) (4wk)
	G2	43.35 (10.46)	40.4 (8.35) (24h)	39.4 (7.87) (4wk)
	Right Cervical Rotation (º)			
Martín-Rodríguez et al, 2019	G1	66.3 (9.50)	61.9 (13.79) (24h)	68.9 (9.12) (4wk)
	G2	59.6 (14.32)	63.3 (10.36) (24h)	67.7 (9.25) (4wk)
	Left Cervical Rotation (º)			
	G1	66.2 (12.15)	65.9 (12.74) (24h)	65.6 (9.91) (4wk)
	G2	61.1 (13.08)	65.2 (11.18) (24h)	64.9 (8.31) (4wk)
	Cervical Rotation (mean calculated)			
	G1	66.25 (10.82)	63.9 (13.26) (24h)	67.25 (9.51) (4wk)
	G2	60.35 (13.7)	64.25 (10.77) (24h)	66.3 (8.78) (4wk)
	Pain (VAS, 0-10)			
	G1	6.51 (0.37)		3.3 (0.24) (2wk)
	G2	6.64 (0.82)		4 (0.85) (2wk)
	G3	6.5 (0.78)		2.96 (0.3) (2wk)
	PPT (kPa)			
Tabatabaieee et al. 2019	G1	111.89 (54.91)		185.34 (29.41) (2wk)
	G2	113.26 (54.91)		158.86 (82.37) (2wk)
	G3	112.08 (81.39)		181.61 (35.30) (2wk)
	Ipsilateral Cervical Lateral Flexion (º)			
	G1	37.84 (0.76)		42.68 (0.47) (2wk)
	G2	37.74 (0.73)		40.69 (0.92) (2wk)
	G3	37.76 (0.87)		43.21 (0.42) (2wk)
	Contralateral Lateral Flexion (º)			
	G1	36.67 (0.64)		43.66 (0.49) (2wk)
	G2	36.4 (0.91)		41.54 (0.68) (2wk)
	G3	36.21 (0.84)		43.8 (0.83) (2wk)
	Cervical Lateral Flexion (mean calculated)			
	G1	37.25 (0.7)		43.17 (0.48) (2wk)
Tabatabaieee et al. 2019	G2	37.07 (0.82)		41.11 (0.8) (2wk)
	G3	36.98 (0.85)		43.5 (0.62) (2wk)
	Ipsilateral Cervical Rotation (º)			

	G1	69.37 (0.86)	73.03 (0.8) (2wk)
	G2	69.1 (0.93)	76.39 (0.55) (2wk)
	G3	68.96 (1.09)	76.22 (0.92) (2wk)
	Contralateral Cervical Rotation (º)		
	G1	71.33 (0.74)	75.68 (0.56) (2wk)
	G2	70.58 (0.47)	73.55 (0.90) (2wk)
	G3	71.19 (0.79)	75.44 (0.64) (2wk)
	Cervical Rotation (mean calculated)		
	G1		
	G2	70.35 (0.8)	74.05 (0.68) (2wk)
	G3	69.84 (0.7)	74.97 (0.72) (2wk)
		70.07 (0.94)	75.83 (0.78) (2wk)
	Pain (NPRS, 0-10)		
	G1	6.4 (1.6)	3.1 (2.4) (4wk)
	G2	6.8 (1.6)	2.7 (2.8) (4wk)
	Disability (NDI)		
	G1	13.4 (4.9)	6.9 (4.9) (4wk)
	G2	16.8 (7.9)	5.6 (4.4) (4wk)
	Cervical Flexion (º)		
	G1	68.0 (6.2)	69.1 (3.7) (4wk)
	G2	57.8 (13.9)	63.3 (8.6) (4wk)
Onat et al. 2019			
	Cervical Extension (º)		
	G1	58.3 (6.9)	59.7 (2.91) (4wk)
	G2	52.2 (9.5)	57.8 (5.4) (4wk)
	Right Cervical Lateral Flexion (º)		
	G1	43.6 (4.2)	45.0 (0.0) (4wk)
	G2	39.0 (7.6)	45.9 (5.6) (4wk)
	Left Cervical Lateral Flexion (º)		
	G1	43.9 (3.6)	45.0 (0.0) (4wk)
	G2	38.8 (7.8)	45.9 (5.6) (4wk)
	Cervical Lateral Flexion (mean calculated)		
	G1	43.75 (7.8)	45 (0.0) (4wk)
	G2	38.9 (7.7)	45.9 (5.6) (4wk)
	Right Cervical Rotation (º)		
	G1	77.5 (10.5)	78.9 (4.6) (4wk)
	G2	65.5 (17.4)	78.1 (6.1) (4wk)
Onat et al. 2019			

	Left Cervical Rotation (º)				
	G1	76.3 (11.3)		79.1 (3.7) (4wk)	
	G2	65.5 (17.1)		78.1 (6.1) (4wk)	
	Cervical Rotation (mean calculated)				
	G1	76.9 (10.9)		79 (4.15) (4wk)	
	G2	65.5 (17.25)		78.1 (6.1) (4wk)	
Ziaeifar et al. 2019	Pain (VAS, 0-10)				
	G1	6.56 (1.63)			2.4 (1.74) (12wk)
	G2	6.23 (1.26)			3.33 (2.22) (12wk)
	Disability (NPQ)				
	G1	2.65 (9.9)		13.17 (11.5) (1wk)	12.4 (8.6) (12wk)
	G2	33 (7.09)		21.39 (12.36) (1wk)	19.32 (12.20) (12wk)
Sukareechai et al. 2019	Pain (NPRS, 0-10)				
	G1	6.8 (1.7)		2.6 (2.2) (3wk)	
	G2	7.2 (1.6)		3.4 (2.1) (3wk)	
	PPT (kPa)				
	G1	537.48 (142.14)		644.09 (128.81) (3wk)	
	G2	519.71 (177.68)		657.41 (168.79) (3wk)	
Arias-Buría et al. 2020	Pain (NPRS,0-10)				
	G1	5.5 (1.4)	4.7 (2.1) (24h)	3.4 (2.2) (4wk)	
	G2	5.7 (1.7)	5.1 (1.9) (24h)	4.8 (2.1) (4wk)	
	Disability (NDI)				
	G1	21.7 (2.2)		16 (2.0) (4wk)	
	G2	20.0 (2.1)		16.0 (1.9) (4wk)	
Valiente Castrillo et al. 2020	Pain (VAS, 0-10)				
	G1	5.79 (1.89)		2.38 (1.85) (4wk)	3.00 (1.73) (12wk)
	G2 (N/A)	5.52 (1.80)		1.17 (1.12) (4wk)	2.47 (2.31) (12wk)
	G3	5.26 (1.46)		3.85 (2.38) (4wk)	3.91 (2.50) (12wk)
	Disability (NDI)				
	G1	17.45 (4.94)		12.00 (5.68) (4wk)	11.0 (6.06) (12wk)
	G2 (N/A)	15.80 (4.62)		7.19 (6.56) (4wk)	7.57 (5.33) (12wk)
	G3	16.78 (5.32)		13.21 (7.26) (4wk)	13.78 (8.78) (12wk)
	Pain (VAS, 0-10)				
	G1	5.01 (1.52)	2.28 (1.58) (48h)	2.26 (1.55) (1wk)	
	G2	4.56 (1.73)	2.04 (1.69) (48h)	1.71 (1.29) (1wk)	
	Disability (NDI)				

	G1	19.64 (7.32)		13.09 (7.45), (1wk)
	G2	22.09 (9.75)		12.27 (8.20) (1wk)
	PPT (kPa)			
	G1	194.17 (54.91)	266.73 (45.09) (48h)	264.77 (50.99) (1wk)
	G2	206.80 (50.99)	376.57 (42.16) (48h)	409.91 (45.11) (1wk)
	Cervical Flexion (º)			
	G1	44.83 (9.85)	48.36 (9.31) (48h)	47.27 (8.41) (1wk)
	G2	44.59 (11.25)	54.73 (6.39) (48h)	51.27 (7.59) (1wk)
García-de-Miguel et al. 2020				
	Cervical Extension (º)			
	G1	50.72 (9.72)	57.32 (7.49) (48h)	58.05 (7.95) (1wk)
	G2	52.81 (13.90)	56.64 (11.37) (48h)	57.68 (11.26) (1wk)
	Cervical Rotation (º)			
	G1	55.19 (13.62)	67.18 (11.00) (48h)	66.32 (9.81)) (1wk)
	G2	60.53 (12.48)	70.95 (8.52) (48h)	70.11 (8.42) (1wk)
	Cervical Lateral Flexion (º)			
	G1	36.44 (5.56)	40.09 (5.30) (48h)	40.70 (4.68)) (1wk)
	G2	37.97 (4.36)	41.04 (5.08) (48h)	41.38 (5.56) (1wk)

BDI; Beck Depression Inventory; PCS: Pain Catastrophism Scale; VAS: Visual Analogue Scale; GDS-SF: Korean version of geriatric depression scales; wk.: Weeks; ROM: Range of Motion; PPT: Pressure Pain Threshold; NDI: Neck Disability Index; NPRS: Numeric Pain Rating Scale; NPQ: Northwick Park Neck Pain Questionnaire; NPDS: Neck Pain and Disability Score.[+] Converted to mean and standard deviation values from median and interquartile range

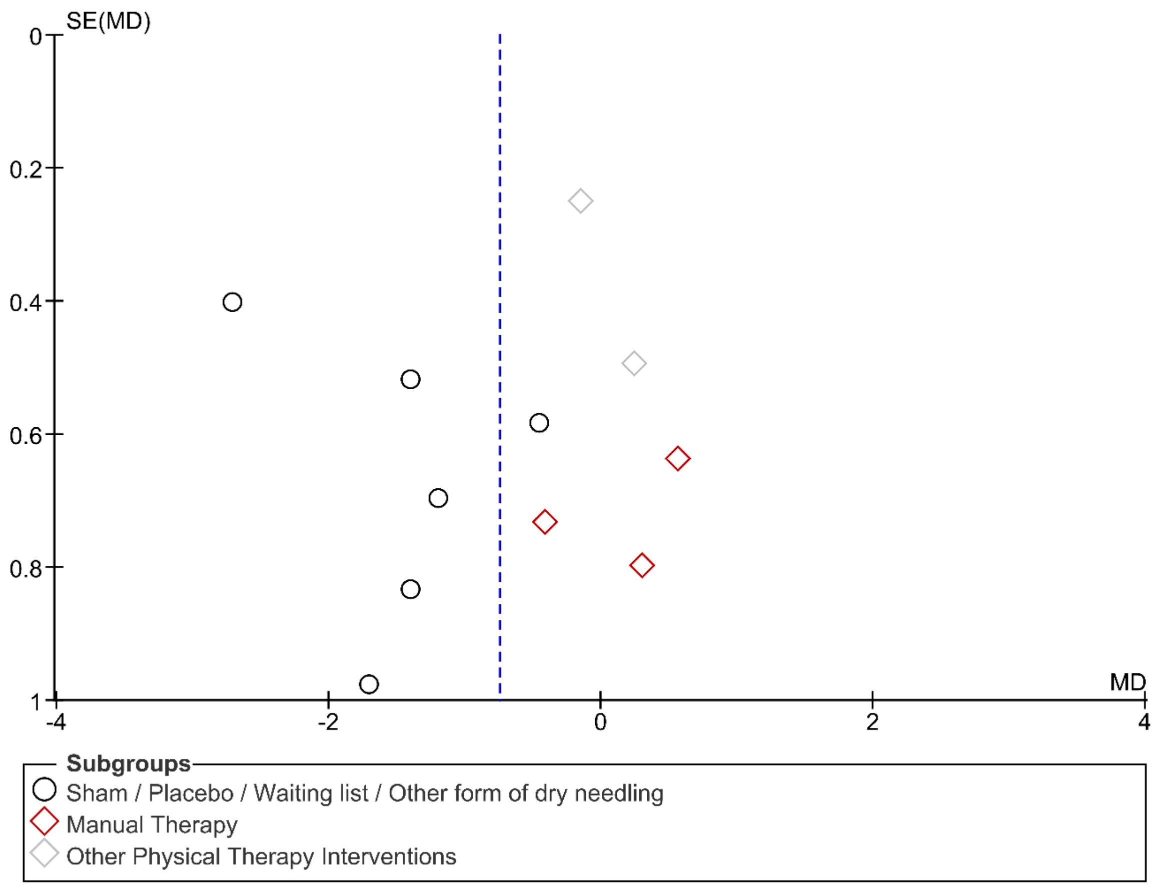

Figure 1. Funnel Plot of the trials (*n* = 11) investigating the immediate effects of dry needling on pain intensity. The funnel plot showed small asymmetry not associated to potential publication bias. SE:Standard error; MD: Mean difference.

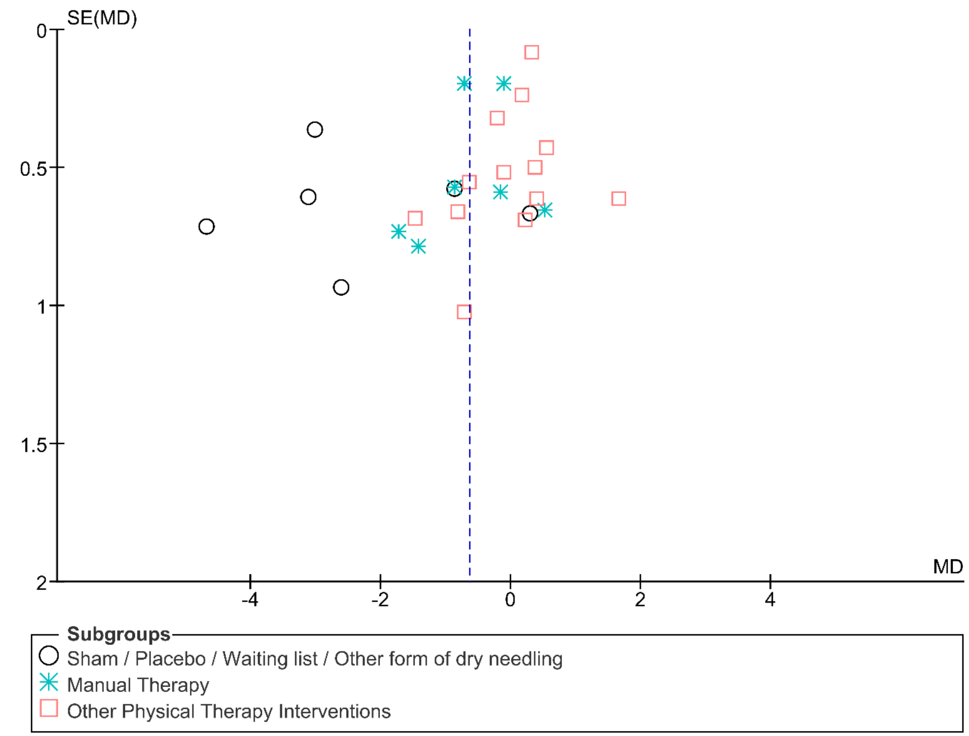

Figure 2. Funnel Plot of the trials (*n*=25) investigating the short-term effects of dry needling on pain intensity. The funnel plot showed small asymmetry not associated to potential publication bias. SE:Standard error; MD: Mean difference.

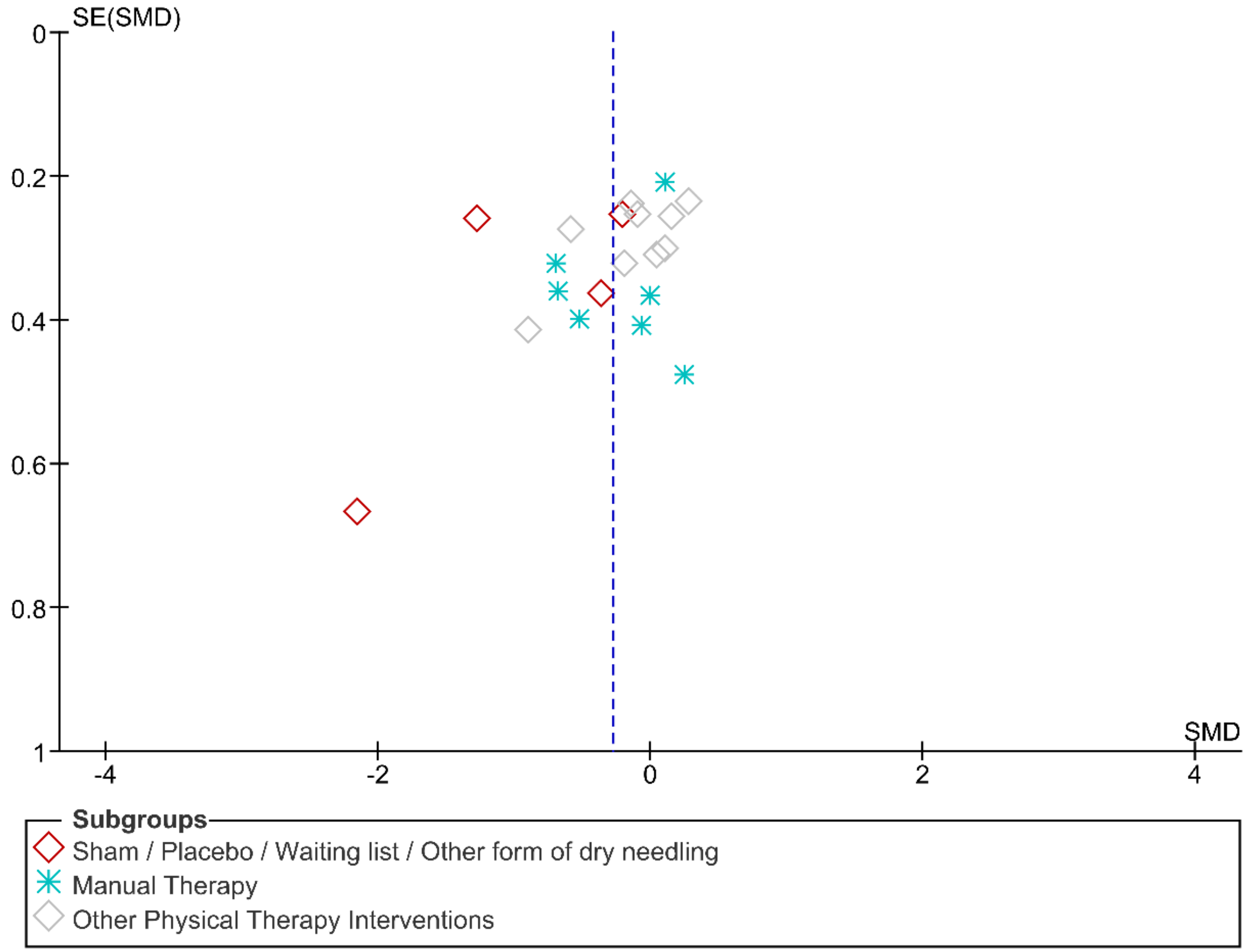

Figure 3. Funnel Plot of the trials (n = 20) investigating the short-term effects of dry needling on pain-related disability. The funnel plot showed asymmetry due to the study by Itoh et al 2007, therefore, it was associated to potential publication bias. The exclusion of this study would tend to a symmetric funnel plot. SE:Standard error; SMD: Standard mean difference.

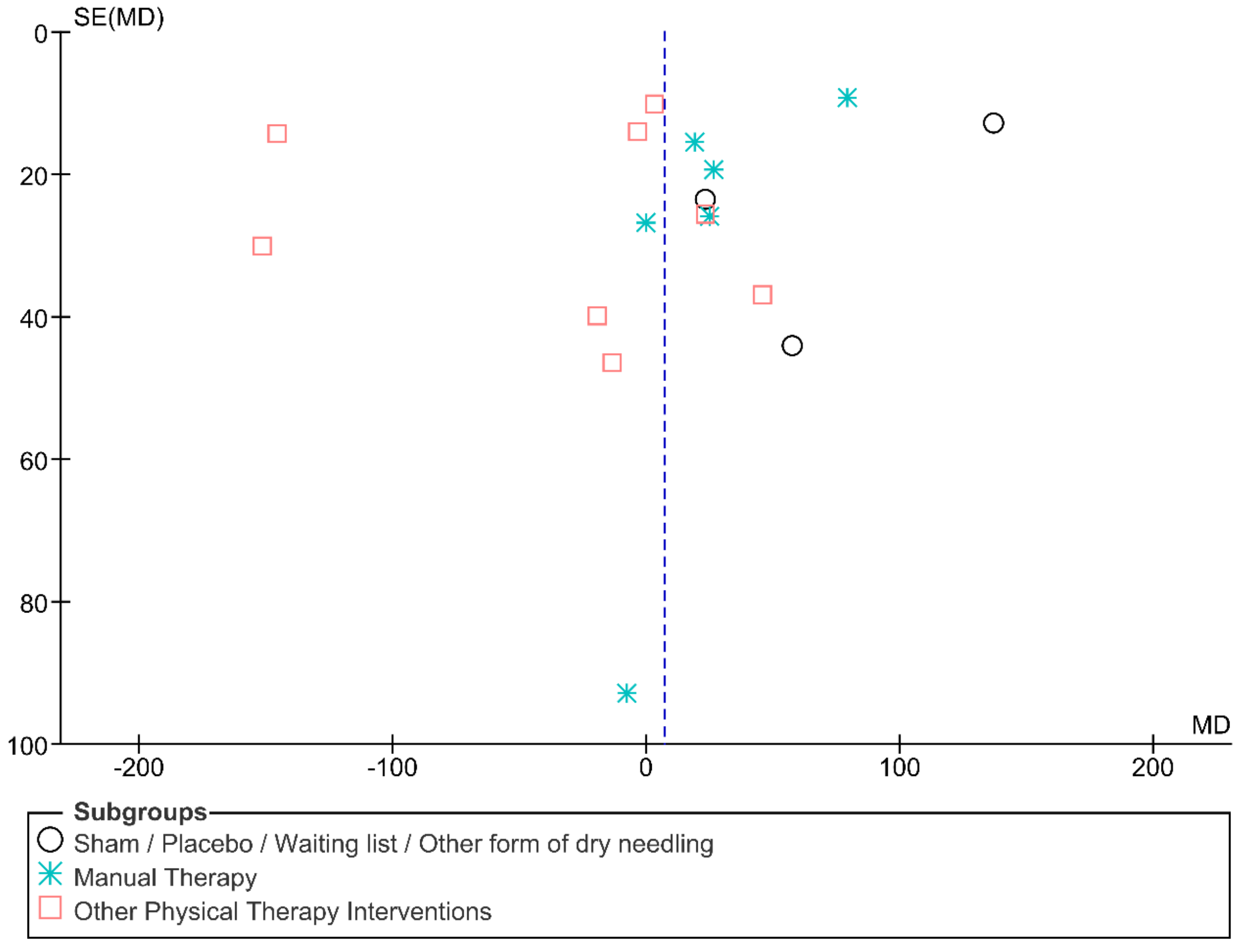

Figure 4. Funnel Plot of the trials (*n* = 17) investigating the short-term effects of dry needling on pressure pain thresholds. The funnel plot showed small asymmetry not associated to potential publication bias. SE:Standard error; MD: Mean difference.

Publisher's Note: MDPI stays neutral with regard to jurisdictional claims in published maps and institutional affiliations.

© 2020 by the authors. Licensee MDPI, Basel, Switzerland. This article is an open access article distributed under the terms and conditions of the Creative Commons Attribution (CC BY) license (http://creativecommons.org/licenses/by/4.0/).
